# European guidelines from the EHTG and ESCP for Lynch syndrome: an updated third edition of the Mallorca guidelines based on gene and gender

**DOI:** 10.1002/bjs.11902

**Published:** 2021-05-26

**Authors:** T T Seppälä, A Latchford, I Negoi, A Sampaio Soares, R Jimenez‐Rodriguez, L Sánchez‐Guillén, D G Evans, N Ryan, E J Crosbie, M Dominguez‐Valentin, J Burn, M Kloor, M von Knebel Doeberitz, F J B van Duijnhoven, P Quirke, J R Sampson, P Møller, G Möslein, the European Hereditary Tumour Group (EHTG) and European Society of Coloproctology (ESCP)

**Affiliations:** 1 Department of Surgery, Helsinki University Hospital, and University of Helsinki, Helsinki, Finland; 2 Department of Surgical Oncology, Johns Hopkins Hospital, Baltimore Maryland, USA; 3 Department of Cancer and Surgery, Imperial College London, UK; 4 St Mark's Hospital, London North West Healthcare NHS Trust, London, UK; 5 Manchester Centre for Genomic Medicine, Division of Evolution and Genomic Sciences, University of Manchester, Manchester University Hospitals NHS Foundation Trust, UK; 6 Division of Cancer Sciences, Faculty of Biology, Medicine and Health, University of Manchester, St Mary's Hospital, Manchester, UK; 7 Centre for Academic Women's Health, University of Bristol, Bristol, UK; 8 Faculty of Medical Sciences, Newcastle Upon Tyne Hospitals NHS Foundation Trust, Newcastle upon Tyne, UK; 9 Pathology and Data Analytics, School of Medicine, University of Leeds, Leeds, UK; 10 Institute of Medical Genetics, Division of Cancer and Genetics, Cardiff University School of Medicine, Heath Park, Cardiff, UK; 11 Department of Surgery, Emergency Hospital of Bucharest, Carol Davila University of Medicine and Pharmacy, Bucharest, Romania; 12 Hospital Prof. Dr Fernando Fonseca, EPE, Lisbon, Portugal; 13 Department of Surgery, Hospital Universitario Virgen del Rocío, Seville, Spain; 14 Colorectal Unit, Department of General Surgery, Elche University General Hospital Elche, Alicante, Spain; 15 Department of Tumour Biology, Norwegian Radium Hospital, Oslo University Hospital, Oslo, Norway; 16 Department of Applied Tumour Biology, Institute of Pathology, University Hospital Heidelberg, Germany; 17 Cooperation Unit Applied Tumour Biology, German Cancer Research Centre, Heidelberg, Germany; 18 Centre for Hereditary Tumours, Bethesda Hospital, Duisburg, Germany; 19 University of Witten/Herdecke, Witten, Germany; 20 Division of Human Nutrition and Health, Wageningen University and Research, Wageningen, the Netherlands

## Abstract

**Background:**

Lynch syndrome is the most common genetic predisposition for hereditary cancer but remains underdiagnosed. Large prospective observational studies have recently increased understanding of the effectiveness of colonoscopic surveillance and the heterogeneity of cancer risk between genotypes. The need for gene‐ and gender‐specific guidelines has been acknowledged.

**Methods:**

The European Hereditary Tumour Group (EHTG) and European Society of Coloproctology (ESCP) developed a multidisciplinary working group consisting of surgeons, clinical and molecular geneticists, pathologists, epidemiologists, gastroenterologists, and patient representation to conduct a graded evidence review. The previous Mallorca guideline format was used to revise the clinical guidance. Consensus for the guidance statements was acquired by three Delphi voting rounds.

**Results:**

Recommendations for clinical and molecular identification of Lynch syndrome, surgical and endoscopic management of Lynch syndrome‐associated colorectal cancer, and preventive measures for cancer were produced. The emphasis was on surgical and gastroenterological aspects of the cancer spectrum. Manchester consensus guidelines for gynaecological management were endorsed. Executive and layperson summaries were provided.

**Conclusion:**

The recommendations from the EHTG and ESCP for identification of patients with Lynch syndrome, colorectal surveillance, surgical management of colorectal cancer, lifestyle and chemoprevention in Lynch syndrome that reached a consensus (at least 80 per cent) are presented.

## Abstract (es)

**Antecedentes:**

El síndrome de Lynch (*Lynch síndrome*, LS) es la predisposición genética más frecuente para el cáncer hereditario, pero es una patología que sigue siendo infradiagnosticada. Recientemente, grandes estudios prospectivos observacionales han permitido comprender mejor la efectividad de la vigilancia mediante colonoscopia y la heterogeneidad del riesgo de cáncer entre genotipos. Se ha reconocido la necesidad de pautas específicas en función de los genes y del género.

**Métodos:**

El Grupo Europeo de Tumores Hereditarios (*European Hereditary Tumour Group*, EHTG) y la Sociedad Europea de Coloproctología (*European Society of Coloproctology*, ESCP) desarrollaron un grupo de trabajo multidisciplinar formado por cirujanos, genetistas clínicos y moleculares, patólogos, epidemiólogos, gastroenterólogos y una representación de pacientes para llevar a cabo una revisión para establecer los grados de evidencia. Se utilizó el formato anterior de las “Guías de Mallorca” para revisar las recomendaciones clínicas. El consenso para las declaraciones de la guía se alcanzó tras tres rondas de votación según el método Delphi.

**Resultados:**

Se elaboraron recomendaciones para la identificación clínica y molecular del LS, manejo quirúrgico y endoscópico del cáncer colorrectal asociado con el LS y medidas preventivas para el cáncer. Se hizo hincapié en los aspectos quirúrgicos y gastroenterológicos del espectro del cáncer. Se aprobaron las recomendaciones de consenso de Manchester para el manejo ginecológico. Se distribuyeron resúmenes para profesionales y el público en general.

**Conclusión:**

Se presentan las recomendaciones de la EHTG y ESCP para la identificación de pacientes con LS, vigilancia colorrectal, manejo quirúrgico del cáncer colorrectal, estilo de vida y quimioprevención en LS en las que se obtuvo consenso (> 80%).

Recommendations for clinical and molecular identification of LS, surgical and endoscopic management of LS‐associated colorectal cancer and preventive measures for cancer were produced. The emphasis was on surgical and gastroenterological aspects of the cancer spectrum.

## Definitions used in these guidelines

- Consensus: at least 80 per cent agreement
- Majority: 50–79 per cent agreement
- Subtotal colectomy: anastomosis is ileosigmoidal
- Total colectomy: anastomosis is ileorectal
- Extended surgery: refers either to subtotal colectomy with ileosigmoidal anastomosis or total colectomy with ileorectal anastomosis, when used to replace an operation that would be oncological standard practice for a sporadic colorectal cancer, because of a pathogenic germline variant
- Amsterdam criteria: these criteria were introduced for uniform classification based on family history and require at least three affected members on the same side of the family in two or more generations, with one being a first‐degree relative of the other two and at least one individual diagnosed before 50 years of age. The Amsterdam I criteria apply to families with three or more colorectal cancers, and the Amsterdam II criteria also include extracolonic tumours: endometrial cancer, cancer of the upper urinary tract and cancer of the small bowel
- Carrier: a person with a germline *path*_*MMR* variant
- Lynch syndrome (LS): the dominantly inherited cancer syndrome caused by the presence of a pathogenic mismatch repair gene variant
- Revised Bethesda guidelines: guidance developed for testing colorectal tumours for microsatellite instability (MSI), when: colorectal or uterine cancer is diagnosed in a patient who is less than 50 years of age; synchronous, metachronous colorectal, or other hereditary non‐polyposis colorectal cancer (HNPCC)‐associated tumours (regardless of age) are present; colorectal cancer with MSI‐high is diagnosed in a patient who is aged less than 60 years; colorectal cancer is diagnosed in one or more first‐degree relatives with an HNPCC‐related tumour, with one of the cancers being diagnosed under age 50 years; colorectal cancer is diagnosed in two or more first‐ or second‐degree relatives with HNPCC‐related tumours, regardless of age
- *Path_MMR*: the pathogenic (disease‐causing) variants of the specified mismatch repair (MMR) gene associated with cancer, including all structural or epigenetic variants of these

## Executive summary of recommendations

Recommendations for the identification of patients with Lynch syndrome (LS), colorectal surveillance, surgical management of colorectal cancer, lifestyle and chemoprevention that reached a consensus (at least 80 per cent) by a combined expert working group from the European Hereditary Tumour Group (EHTG; former Mallorca Group) and the European Society of Coloproctology (ESCP) are presented in *Table* *1*. In addition, there was agreement to endorse the Manchester consensus statement for gynaecological cancer in LS^1^. The summary of recommendations for healthcare professionals is as follows.

**Table 1:** Recommendations that achieved consensus based on GRADE

	Strength of recommendation	% of voters agreeing
**Identification of LS**		
Amsterdam criteria and/or revised Bethesda criteria are not sufficient to guide tumour testing owing to low sensitivity in detection of patients with LS	Strong*	86
All colorectal cancers should be tested by MMR (MLH1, MSH2, MSH6, PMS2) immunohistochemistry or MSI testing (followed by possible *MLH1* hypermethylation testing) to screen for LS	Strong*	91
Immunohistochemistry performed on preoperative colorectal cancer biopsies is at least as accurate as that performed on resection specimens	Strong*	81
**Colorectal surveillance**		
For *path_MLH1*, *path_MSH2* and *path_MSH6* carriers, 2‐ or 3‐yearly colonoscopic surveillance is recommended^‡^	Strong*	75
For *path_PMS2* carriers, colonoscopic surveillance should be performed to reduce mortality and incidence of colorectal cancer	Strong*	82
For *path_PMS*2 carriers, 5‐yearly surveillance may be considered	Weak^†^	80
For patients with LS with a history of CRC and segmental colectomy, biennial colonoscopies should be performed	Strong*	88
For patients with LS with a history of CRC and segmental colectomy, biennial rectosigmoidoscopies should be performed	Strong*	88
There is no evidence at the moment to support different surveillance colonoscopy intervals for men and women	Strong*	100
Chromoendoscopy is equivalent to high‐definition white‐light endoscopy in specialist centres. It may be an adjunct to be considered in the absence of high‐definition endoscopy or in centres with lower adenoma detection rates	Weak^†^	92
If bowel preparation is not entirely adequate, a repeat procedure at 1 year is recommended. If the bowel preparation is completely inadequate or the examination incomplete, an immediate repeat colorectal surveillance procedure should be requested (within next 6 weeks)	Weak, based on very low‐quality evidence (expertopinion)	85
Age at onset of surveillance colonoscopy should be stratified according to genotype	Strong*	100
For *path_MLH1* or *path_MSH2* carriers, surveillance colonoscopies should be initiated at the age of 25 years	Moderate^†^	94
For *path_MSH6* or *path_PMS2* carriers, surveillance colonoscopies should be initiated at the age of 35 years	Moderate^†^	93
Age at onset of surveillance should not be stratified by gender	Moderate*	88
**Surgical management of colorectal cancer**		
For a *path_MLH1* or *path_MSH2* carrier with a first colonic cancer, extended surgery with ileosigmoidal/ileorectal anastomosis is preferable to standard resection to reduce the risk of metachronous CRC	Strong*	82
For a *path_MSH6* or *path_PMS2* carrier with a first colonic cancer, standard/segmental colonic resection should be offered	Weak*	80
For a *path_MMR* carrier with a metachronous colonic cancer, the surgical treatment can be extended surgery with ileorectal/ileosigmoidal anastomosis.	Weak, based on very low‐quality evidence	93
A decision on extended colorectal surgery for CRC should not be based on dMMR immunohistochemistry (loss of *MLH1*, *MSH2*, *MSH6* or *PMS2*) and BRAF staining/*MLH1* hypermethylation from the preoperative endoscopic biopsy only	Strong*	94
For a *path_MMR* carrier, the surgical treatment of a primary rectal cancer (occurring as the first colorectal cancer) should be standard resection (anterior resection or APR)	Strong*	92
In a young *path_MMR* carrier with a rectal cancer and a synchronous neoplasia or a personal preference, extended surgery can be considered	Weak, based on very low‐quality evidence	86
Ileoanal pouch surgery (in agreement with ECCO guidelines for pouch surgery in ulcerative colitis) should be performed in highly specialized colorectal surgical units	Moderate^†^	92
For endoscopically non‐removable polyps with advanced histology, an oncological approach is recommended, as for gene‐specific treatment for carcinoma	Strong*	93
Prophylactic colorectal surgery in the absence of neoplastic lesions in the colorectum is not recommended for *path_MMR* carriers based on their pathogenic variant‐related risk only	Strong*	97
**Lifestyle and chemoprevention**		
*Path_MMR* carriers should be advised that smoking increases the risk of colorectal cancer	Weak^†^	100
*Path_MMR* carriers should be advised that obesity increases the risk of colorectal cancer	Weak^†^	100
*Path_MMR* carriers should be advised that physical activity reduces the risk of colorectal cancer	Weak^†^	90
*Path_MMR* carriers should be advised that alcohol consumption increases the risk of colorectal cancer	Weak^†^	82
*Path_MMR* carriers should be advised that there is a high probability that daily aspirin will reduce cancer risk	Weak#	90
Patients with *path_MMR* should be advised that there is a high probability that daily aspirin will reduce their cancer risk	Weak*	100
The recommended aspirin dose should be a minimum of 75–100 mg daily. This dose should be increased for people with above‐average body mass	Weak^†^	93

*Based on moderate‐quality evidence; ^†^based on low‐quality evidence. ^‡^Consensus not reached between 2‐ and 3‐year interval. GRADE, Grading of Recommendations, Assessment, Development and Evaluation; LS, Lynch syndrome; MMR, mismatch repair; MSI, microsatellite instability; CRC, colorectal cancer; dMMR, deficient mismatch repair; APR, abdominoperineal resection; ECCO, European CanCer Organisation.

**Table 2:** Overview of chromoendoscopy studies in Lynch syndrome

Reference	Study outcomes
Brown *et al*.^24^	CE yielded more people with at least one neoplastic lesion (OR 1·53, 95 per cent c.i. 1·31 to 1·79). However, the authors felt the evidence base was too small
Kamiński *et al*.^25^	The study strongly recommended the routine use of high‐definition pancolonic CE in patients with known or suspected LS (conventional CE, NBI, i‐SCAN), but acknowledged the low‐quality evidence
Lecomte *et al*.^26^	A small series reported that CE improved the adenoma detection rate in tandem colonoscopy studies
Stoffel *et al*.^27^	Patients underwent white‐light colonoscopy and were then randomized to CE *versus* intensive inspection (withdrawal time of at least 20 min). CE was superior to standard white‐light examination but not significantly different from intensive inspection
Rahmi *et al*.^28^	Patients underwent tandem colonoscopy with white light then CE. CE was superior, identifying 32 of 78 patients with one or more adenomas *versus* 18 of 78 (*P* < 0·001) and an additional adenoma in 24 of 78 (31%).
van Rijn *et al*.^29^	A 20% adenoma miss rate in average‐risk cohorts was reported in a systematic review of tandem white‐light/CE studies
East *et al*.^30^	The authors reported a significantly increased adenoma detection rate with NBI. However, there was no randomization of the order in which NBI and white‐light colonoscopy were undertaken in this study, which is a major methodological criticism
Bisschops *et al*.^31^	An increased adenoma detection rate was reported with the use of i‐SCAN *versus* high‐definition white‐light colonoscopy in 61 patients with LS. Patients were randomized to i‐SCAN or white light first
Hüneburg *et al*.^32^	In a comparison of white light with CE and virtual chromoendoscopy, CE was found to be significantly better than NBI and standard white‐light colonoscopy
Rondagh *et al*.^33^	Findings in patients with and without LS were compared. LS adenomas were more likely to be non‐polypoid (43 *versus* 17%; OR 3·6, *P* < 0·001). This was particularly so for those in the proximal colon (58 *versus* 16%; OR 6·93, *P* < 0·001). Advanced histology was more likely to be found in non‐polypoid adenomas in patients with LS than those without (4 of 5 *versus* 5 of 12). Serrated lesions were more likely to be non‐polypoid in LS (49 *versus* 20%; OR 3·57, *P* < 0·001)

CE, chromoendoscopy; OR, odds ratio; NBI, narrow‐band imaging; i‐SCAN, postprocessing software filter technology; LS, Lynch syndrome.

### General information

- LS is caused by inherited malfunction in one of the four MMR genes named *MLH1*, *MSH2*, *MSH6* and *PMS2*. The malfunctioning variants are referred to as *path_MLH1*, *path_MSH2*, *path_MSH6* and *path_PMS2*. People carrying inherited *path_MMR* or inherited epigenetically silenced variants of these genes are referred to as carriers.
- Carriers have an increased risk of developing colorectal, endometrial, ovarian, urinary tract, prostate and other cancers, depending on which gene is malfunctioning. Therefore, consideration to the responsible gene and gender should be given where a pathogenic variant has been identified.
- The average risk for cancer stratified by gene, organ, gender and age is available at http://www.PLSD.eu. This resource allows personalized genetic counselling to support decision‐making.
- The guidelines presented here are designed to empower patients and clinicians to enable informed and individualized decision‐making; the authors recognize that there is no universal approach to the care of those who carry *path_MMR* and have LS, and therefore personalized care is critical.

### Identification of carriers

- All colorectal and endometrial cancer should be tested for evidence of MMR deficiency to screen for LS. If not limited by resources, all those with colorectal or endometrial cancer can undergo direct germline testing for *path_MMR*.
- The established clinical criteria may be used for selecting people without cancer to be genetically tested.

### Surveillance for colorectal cancer

- Colonoscopy is recommended every 2 or 3 years for *path*_*MLH1*, *path*_*MSH2* and *path*_*MSH6* carriers, unless they have had colorectal cancer before, after which biennial colonoscopy is recommended.
- *Path*_*PMS2* carriers may be considered for 5‐yearly colonoscopy.
- Colonoscopy surveillance is recommended starting at age 25 years for *path*_*MLH1* and *path*_*MSH2* carriers, and at age 35 years for *path*_*MSH6* and *path*_*PMS2* carriers.
- The recommended surveillance for colorectal cancer does not differ between men and women.

### Surveillance, management and prevention of gynaecological cancer

- The Manchester International Consensus Group^1^ recommendations for the surveillance, management and prevention of gynaecological cancers in LS are endorsed.

### Surgical management of colorectal cancer

- Extended surgery is recommended for *path*_*MLH1* and *path*_*MSH2* carriers at the time of first diagnosis of a colonic cancer.
- Removing part of the colon or rectum in the absence of cancer or endoscopically non‐removable polyps is not generally recommended for *path_MSH6* and *path_PMS2* carriers.

**Fig. 1:**
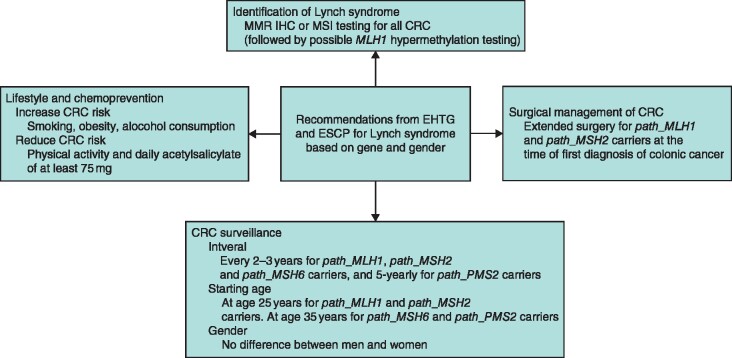
Summary of core content of the recommendations MMR, mismatch repair; IHC, immunohistochemical; MSI, microsatellite instability; CRC, colorectal cancer; EHTG, European Hereditary Tumour Group; ESCP, European Society of Coloproctology.

### Lifestyle and chemoprevention

- Smoking and obesity increase the risk of colorectal cancer in carriers.
- Alcohol consumption increases the risk of colorectal cancer in carriers.
- Physical activity reduces colorectal cancer risk in carriers.
- Daily acetylsalicylic acid of at least 75 mg reduces cancer risk in carriers.

Recommendations for professionals have been summarized in *Fig*. *1* and *Table S1* (supporting information). Universal laboratory screening for upper urinary tract carcinomas to identify LS was approved by a majority (73 per cent) but did not reach consensus. A summary of recommendations for patients (layperson summary) is presented in *Table S2* (supporting information).

## Introduction

LS (OMIM #120435) is the most common dominantly inherited cancer syndrome but is often not recognized^2^^,^^3^. The prevalence of *path_MMR* carriers has been estimated to be around one in 300, that is 2·5 million people in Europe alone^4^.

Carriers are at increased risk of developing cancers, including colorectal, endometrial, ovarian, stomach, pancreatic, small bowel, biliary tract, urinary tract, brain and skin cancer. In LS, these cancers may occur much earlier in life than their sporadic counterparts, but older age of onset is not infrequent, and penetrance and expression vary by gene and gender from very high to immeasurable. The International Society for Gastrointestinal Hereditary Tumours (InSiGHT), through the use of its international database (http://insight‐database.org/) and expertise, categorizes MMR gene variants as being pathogenic (class 5) or likely to be pathogenic (class 4) (or as being of uncertain significance, likely not pathogenic or not pathogenic). This information can then be used to inform patient‐centred counselling in consultation with the Prospective Lynch Syndrome Database (PLSD) (http://www.plsd.eu). Potentially modifiable factors include acetylsalicylic acid (aspirin) prophylaxis and lifestyle. Typically, LS‐associated cancers have significantly better prognoses than sporadic cancers affecting the same organs, reflecting biological differences, including the marked immune responses that characterize LS cancers^5^. Patients with LS who are within cancer surveillance programmes benefit from a stage shift and earlier cancer diagnosis and usually survive their first cancers. Despite surveillance, those with LS can go on to develop metachronous cancers, with service planning implications^6^.

**Fig. 2:**
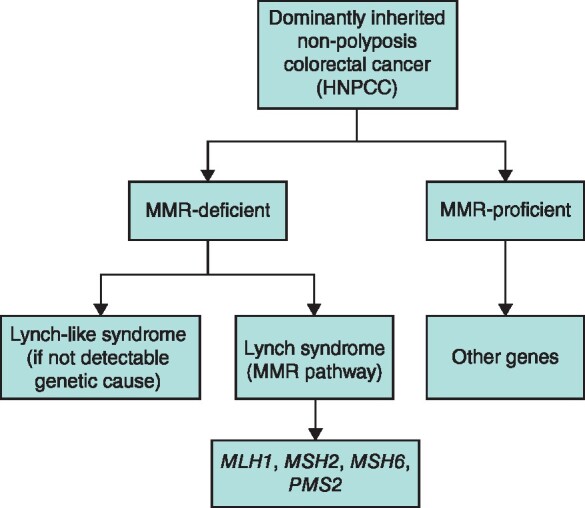
Clinical classification of hereditary non-polyposis colorectal cancer and causal genes^7^ HNPCC, hereditary non-polyposis colorectal cancer; MMR, mismatch repair.

Carriers are identified by demonstration of a class 5 or 4 germline *path_MMR* variant. HNPCC refers to a family history indicating dominantly inherited colorectal cancer, which was later divided to specify several distinct, inherited, cancer syndromes that include colorectal cancer and other cancers (*Fig*. *2*)^7^. Previously published guidelines^8^^,^^9^, for clinical management of LS were based on retrospective studies and made uniform management recommendations, regardless of which gene was involved.

## Methods

### Guideline group

In 2007, a group of European experts (the Mallorca Group) published guidelines for the clinical management of LS^10^ that were revised in 2013^8^. The present second revision was conducted by a combined expert working group from the EHTG (former Mallorca Group) and ESCP, and was based on previously selected clinical questions. The group consisted of surgeons, clinical and molecular geneticists, pathologists, epidemiologists, gastroenterologists and a patient representative. If a particular specialty was not represented, specialists outside the group were consulted. It was decided that the update format of the manuscript would be via a continuous update process to be undertaken by the EHTG (living guidance).

### Scope of updated guidelines

This update is based on the results from new prospective studies that reported the average risk of cancer in carriers by organ, by age, by gene and by gender. It covers the screening, diagnosis and management of LS, and is targeted for patients with this condition. Its aim is to gather the most up‐to‐date evidence on the management of LS. Furthermore, this guideline addresses the clinical questions raised in previous versions, and updates recommendations for the clinical management of carriers. Specifically, the guideline details the treatment and prevention of cancers based on gene and gender, and identifies areas requiring more research. This document is directed to patients of both genders and all age groups in which the diagnosis of LS is suspected or confirmed. The recommendations are directed to all patients with the condition, irrespective of severity or co‐morbidities.

### Literature search

A PICO (Patients, Intervention, Comparison, Outcome) model structure was created for each area of interest, based on previously published template questions^8^. A systematic literature search was performed using the PubMed database and the Cochrane Database of Systematic Reviews, and manual searches of relevant articles up until November 2018. The following Medical Subject Heading (MeSH) terms were used: ‘hereditary nonpolyposis colorectal cancer’[All Fields] OR ‘Lynch syndrome’[All Fields]; 3893 articles were identified. The titles were screened and relevant articles written in English were reviewed, and the level of evidence was graded as high, moderate, low or very low, according to GRADE (Grading of Recommendations, Assessment, Development and Evaluation) criteria (http://www.gradeworkinggroup.org/) (*Table S3*, supporting information).

### Timeline and meetings

During the working group meetings (3 meetings and several teleconferences), the outcomes of the literature search were discussed in detail. PICOs are available in *Appendix**S1* (supporting information) and Delphi round results in *Table S4* (supporting information).

Suggestions for guidance statements emerging from the literature review were formulated and tested through Delphi model‐based voting rounds. The first Delphi round took place as an online questionnaire on SurveyMonkey® ( https://www.surveymonkey.co.uk) in week 38 of 2018. The stakeholders were identified from the membership of the EHTG to provide multidisciplinary expertise. The statements were thereby revised for a second Delphi round via live voting which took place in week 39 of 2018, among participants at the Third Annual Meeting of the EHTG in Nice, France. The statements were revised again, and a third Delphi round was conducted by SurveyMonkey® with voting in weeks 32–34 of 2019, again among multidisciplinary stakeholders identified by the EHTG. The most recent results from the PLSD studies were disclosed to all participants before voting, but were only published later^11^. The threshold for reaching consensus in the Delphi votes was set at 80 per cent.

The AGREE reporting checklist was used to guide the reporting of the process (*AppendixS1*, supporting information). An external review of the statements was undertaken before the third Delphi round, focusing mainly on the language of the statements communicating the strength of the recommendation based on the quality of evidence.

## Question 1: how can the identification of Lynch syndrome be improved?

LS carcinomas of the large bowel are associated with a highly cellular, medullary or mucinous appearance, with a marked increase in the number of intratumoral and peritumoral lymphocytes, a pushing border with a low frequency of budding and low stromal content. They have a lower rate of nodal spread. Although these phenotypic features are in keeping with aberrant MMR protein expression, they are not specific for LS.

The clinical criteria for preselection of people for genetic testing (Amsterdam II^12^ and revised Bethesda^13^ criteria) are insensitive and lack specificity in identifying carriers (*Table S5*, supporting information); testing of incident colorectal cancer cases has been recommended for over a decade. In parallel with the present work, a consensus guideline meeting on gynaecological cancer in LS recommended screening for LS in patients with endometrial and ovarian cancer (and also *BRCA1/2* screening in ovarian cancer). Furthermore, identification of carriers provides the opportunity for cascade testing of relatives, which has the benefit of identifying healthy carriers. These approaches have been shown to be cost‐effective^14^^,^^15^.

Laboratory screening of patients with upper urinary tract cancer to identify LS was agreed by a majority (73 per cent) but did not reach consensus.

### Conclusions and recommendations

- All colorectal cancers should be tested by MMR (MLH1, MSH2, MSH6, PMS2) immunohistochemistry or MSI testing (followed by possible *MLH1* hypermethylation testing) to screen for LS.
- Patients with endometrial or ovarian cancer should be screened by immunohistochemistry or DNA testing as described in the Manchester consensus statement.

## Question 2: what is the optimal colorectal surveillance protocol for Lynch syndrome?

### Subquestion 1: should colonoscopic surveillance be performed?

Data from largely observational studies conducted since the 1990s have supported the role of colonoscopic surveillance in patients with LS. Järvinen and colleagues^16^^,^^17^ reported on the outcomes of a cohort of patients with LS who underwent colonoscopic surveillance and compared them with a group who did not, and observed a 62 per cent reduction in colorectal cancer in the surveillance group. Similar observations were made in other studies^18–20^. Altogether, colonoscopic surveillance seems to lead to a 60–72 per cent decrease in colorectal cancer mortality. More recent data from prospective observational studies have questioned the previously reported benefits in earlier studies^16,17,20^ of comparative colorectal cancer incidence. Specifically, nearly half of *path_MLH1* and *path_MSH2* carriers develop colorectal cancer under surveillance^6,21–23^, and the benefits of colonoscopic surveillance are mainly to improve survival by earlier detection compared with no surveillance.

#### Conclusions and recommendations

- There was consensus agreement that for all *path_MMR* carriers colonoscopic surveillance should be performed to reduce mortality and incidence of colorectal cancer.

### Subquestion 2: should chromoendoscopy/virtual chromoendoscopy be performed?

Chromoendoscopy (CE) uses contrast dyes (usually indigocarmine) to highlight the mucosal surface contours and enhance visualization. In virtual CE, filtering technology is built into the colonoscope system, which alters the white‐light image to highlight mucosal surface architecture and capillary pattern. Results with use of CE and virtual CE are summarized in *Table 2*^24–33^. These studies are largely methodologically flawed, which limits the conclusions that can be drawn. CE may be equivalent to high‐quality white‐light examination with prolonged withdrawal. However, given the conflicting results of studies, and the methodological flaws of most published studies, CE may still be a helpful adjunct to be considered in colonoscopic surveillance in LS.

#### Conclusions and recommendations

- Chromoendoscopy is equivalent to high‐definition white‐light endoscopy in specialist centres. It may be an adjunct to be considered in the absence of high‐definition endoscopy or in centres with lower adenoma detection rates.

### Subquestion 3: what is the appropriate colonoscopic surveillance interval?

In studies from the PLSD published by Møller and colleagues^21^, the interval between last surveillance colonoscopy and colorectal cancer was analysed. Of 145 colorectal cancers, 100 were diagnosed more 2 years after the last colonoscopy (interval after colonoscopy range 0–125 months). On the other hand, the high incidence of colorectal cancers in the PLSD despite colonoscopic surveillance was not due to the inclusion of a large cohort of Finnish *path*_*MLH1* carriers for whom a 2–3‐year interval between colonoscopies had been recommended^34^. The cumulative incidence of colorectal cancer or stage at detection did not differ between 1‐yearly, 2‐yearly and 2–3‐yearly colonoscopic surveillance strategies in a study of 2747 *path_MLH1*, *path_MSH2* and *path_MSH6* carriers who were followed up for a total of 23 309 years by 16 327 colonoscopies^23^.

Gathering evidence suggests that carcinogenesis in *path_PMS2* is predominantly via the adenomatous pathway^35^, and that the cancer risk is substantially lower than that for other LS genotypes, especially under colonoscopic surveillance^6^.

There is no reported substantial difference in lifetime incidence of colorectal cancer between that estimated by retrospective segregation analyses over three generations^36–38^ and that in the PLSD report^6^ on the effects of colonoscopy in preventing colorectal cancer as advocated by recent international guidelines (*Table* *3*).

**Table 3:** Studies describing the cumulative incidence of colorectal cancer in patients with Lynch syndrome

		70‐year cumulative incidence (%)		
Reference	Gender	*MLH1*	*MSH2*	*MSH6*	*PMS2*
Bonadona *et al*.^38^	Both	41	48	12	
Dowty *et al*.^36^	Men	34	47		
	Women	36	37		
ten Broeke *et al*.^37^	Men				13*
	Women				12*
PLSD^6^	Men	53	42	18	10
	Women	44	46	20	10

*Cumulative risk at 80 years. PLSD, Prospective Lynch Syndrome Database.

#### Conclusions and recommendations

- For *path_MLH1*, *path_MSH2* and *path_MSH6* carriers, 2‐ or 3‐yearly colonoscopic surveillance is recommended (consensus not reached between 2 and 3 years).
- For *path_PMS2* carriers, 5‐yearly surveillance may be considered.
- For patients with LS with a history of colorectal cancer and segmental or subtotal colectomy, biennial colonoscopies should be performed.
- There is no evidence at the moment to support different surveillance colonoscopy intervals for men and women.
- If bowel preparation is not entirely adequate, a repeat procedure at 1 year is recommended.
- If the bowel preparation is completely inadequate or the examination incomplete, an immediate repeat colorectal surveillance procedure should be requested (within next 6 weeks).

### Subquestion 4: at what age should surveillance colonoscopy be initiated?

A number of studies have confirmed that the risk of developing colorectal cancer before the age of 25 years is very low (*Table* *4*)^6^^,^^8^^,^^21^^,^^22^^,^^37–48^.

**Table 4:** Overview of studies about age and risk of developing colorectal cancer in Lynch syndrome

Reference	Study outcomes
de Jong *et al*.^39^	Only two of 246 patients (0·8%) with LS developed CRC before the age of 20 years and another two between age 20 and 25 years
Hendriks *et al*.^40^ Quehenberger *et al*.^41^ Hampel *et al*.^42^ Jenkins *et al*.^43^	These studies confirmed that the risk of developing CRC before the age of 25 years is very low
Vasen *et al*.^8^ Cairns *et al*.^44^ Rubenstein *et al*.^45^	These previous guidelines recommended starting surveillance colonoscopy at age 20–25 years.
Jenkins *et al*.^46^	This meta‐analysis for *path_MLH1* and *path_MSH2* carriers questioned whether the initiation of surveillance colonoscopy was justified before the age of 30 years
Giardiello *et al*.^47^	US multisociety task force guidelines recommended starting surveillance colonoscopy at 20–25 years (or 2–5 years younger than youngest affected if aged <25 years), but to consider starting at 30 and 35 years for *path_MSH6* and *path_PMS2* carriers respectively
ten Broeke *et al*.^37^^,^^48^	The study described a series of 377 patients with *path_PMS2* and the observed median age at first CRC was 52 (range 26–86) years. The authors also noted gender differences in CRC risk. The cumulative risk in men aged <40, 40–49, 50–59 and 60–69 years was 1·3, 4·6, 7·1 and 18·8% respectively. The corresponding cumulative risks by age in women were 0·5, 0·9, 4·7 and 10·5%. It was recommended that starting colonoscopy surveillance could be deferred until age 30 years for both genders. ten Broeke *et al*. also published a large series of patients with *path_PMS2,* and reported only a 2–3 times increased cumulative incidence of CRC under surveillance compared with the general population. This is about the same level of CRC risk as when there is a positive family history of the disease but genetic testing for LS (and other hereditary CRC syndromes) proves negative
Bonadona *et al*.^38^	This nationwide French study of patients with LS supports a genotype–phenotype correlation. There were no *path_PMS2* carriers in this cohort. Overall, the cumulative CRC risk by 70 years was 38% in men and 31% women. When analysed by genotype, the cumulative CRC risks were 46% for *path_MLH1*, 48% for *path_MSH2* and 12% for *path_MSH6*. Median age at diagnosis of CRC also varied by genotype: 45 (range 15–90) years for *path_MLH1*, 44 (16–95) years for *path_MSH2* and 54 (24–85) years for *path_MSH6*
Møller *et al*.^6^^,^^21^^,^^22^	In the 2017 PLSD report addressing incidence and survival of first cancers in patients with LS undergoing surveillance, the cumulative CRC incidence to age 70 years was 46% for *path_MLH1*, 35% for *path_MSH2*, 20% for *path_MSH6* and 0% for *path_PMS2*. Overall, when incidence was analysed by gender, no difference was observed between men and women. The recent report (2018) from the PLSD described cancer risks and survival up to age 75 years, analysing the data both by gene and gender. The cohort included 3119 patients with 24 475 observation years. The previously described genotype‐dependent penetrance was confirmed; the cumulative CRC risk to age 75 years by genotype was: 46% for *path_MLH1*, 43% for *path_MSH2*, 15% for *path_MSH6* and 0% for *path_PMS2* (although there were only 124 *path_PMS2* carriers in this cohort, so no firm conclusions could be drawn regarding this subgroup). *Path_MSH6* has a lower risk of early‐onset cancer; the cumulative CRC risk at age 40 years was 12% for *path_MLH1*, 9% for *path_MSH2* and 0% for *path_MSH6*. There were no consistent gender differences for CRC within each genotype group

LS, Lynch syndrome; CRC, colorectal cancer; PLSD, Prospective Lynch Syndrome Database.

The risk of a *path_PMS2* carrier developing cancer at young age is low, and the median age for *path_PMS2*‐related first colorectal cancers is 52 years^47^. Based on PLSD studies, *path_MSH6* carriers also have a lower risk of early‐onset cancer; the cumulative colorectal cancer risk at age 40 years was 12 per cent for *path_MLH1*, 9 per cent for *path_MSH2* and 0 per cent for *path_MSH6*. There is no convincing evidence to show that the age of onset is different between men and women.

#### Conclusions and recommendations

- For *path_MLH1* or *path_MSH2* carriers, surveillance colonoscopies should be initiated at the age of 25 years.
- For *path_MSH6* or *path_PMS2* carriers, surveillance colonoscopies should be initiated at the age of 35 years.
- Age at onset of surveillance should not be stratified by gender.

## Question 3: what is the effectiveness of surveillance for other cancers?

The individual cumulative risk of cancer in any organ for the remaining lifetime of a patient with LS may be obtained by indicating their age, gender and genetic variant using http://www.plsd.eu/. There is no demonstrated benefit of surveillance for incidence or survival of gastric cancer, small bowel cancer, pancreatic cancer, urinary tract cancer, prostate cancer or breast cancer in *path_MMR* carriers (*Table* *5*)^6^^,^^49–56^.

**Table 5:** Studies describing cumulative risk and surveillance outcome for gastric, small bowel, pancreatic, urinary tract, prostate and breast cancer in Lynch syndrome

Reference	Study outcomes
**Gastric**	
Møller *et al*.^6^	There is a relative increase in incidence of 8·9 for *path_MLH1* and 9·7 for *path_MSH2* carriers compared with the general population
Park *et al*.^49^	Geographical differences may be significant, with this report from Korea showing a higher lifetime risk
Capelle *et al*.^50^	The Dutch Gastric Cancer Registry study revealed that the majority of gastric cancers in this patient population is of the intestinal type (62%)
Renkonen‐Sinisalo *et al*.^51^	The study collected data on 73 mutation‐positive patients with a mean age of 49 years. In this group, an upper endoscopy for surveillance did not identify any neoplastic changes
**Small bowel**	
Møller *et al*.^6^	There is a relative increase in incidence of this type of cancer of 64·7 for *path_MLH1* carriers
ten Kate *et al*.^52^	The study reported distribution predominantly in the duodenum and jejunum
Saurin *et al*.^53^	The authors reported on 35 asymptomatic patients undergoing capsule endoscopy, which identified lesions in three patients. Of these, one had a jejunal adenocarcinoma and two had adenomas with low‐grade dysplasia
Haanstra *et al*.^54^	The authors examined the effect of video capsule endoscopy in the detection of small bowel neoplasia in 200 asymptomatic patients. In this group, there were significant findings in two patients, who were diagnosed with adenocarcinoma (1 patient) and adenoma (1). An adenocarcinoma diagnosed 7 months after a negative capsule endoscopy was considered a missed lesion. Two years later, 155 of the initial 200 patients underwent a second video capsule endoscopy. Findings leading to further endoscopies with gastroduodenoscopy or balloon‐assisted endoscopy were seen in 11% of patients but none proved to be neoplasia
**Pancreatic**	
Møller *et al*.^6^	The relative risk of pancreatic cancer is 7·8 for *path_MLH1* carriers
Canto *et al*.^55^	In this large study of screening in 354 patients at high risk of pancreatic cancer, a survival benefit was identified for patients diagnosed during screening. This study used endoscopic ultrasonography, MRI and CT for screening. However, no patients with LS were diagnosed with a tumour
**Urinary tract**	
Møller *et al*.^6^	There is a relative increase in incidence of bladder cancer of 4·1 for *path_MLH1* and 8·1 for *path_MSH2* carriers. There is also a relative increase in incidence of ureter and kidney cancer of 3·5 for *path_MLH1* and 13·7 for *path_MSH2* carriers
Myrhøj *et al*.^56^	The National Danish HNPCC registry assessed a strategy of screening with urinary cytology. In this study of 977 individuals, including 263 with confirmed *path_MMR* variants, urine cytology had a sensitivity of 29%, with a specificity of 96%. Five of 14 cancers that occurred were interval cancers not detected by screening
**Prostate**	
Møller *et al*.^6^	There is a relative increase in incidence of this type of cancer of 3·2 for *path_MSH2* carriers
IMPACT study (http://impact.icr.ac.uk/)	The study is currently recruiting carriers of *path_MLH1*, *path_MSH2* and *path_MSH6*, and those who have tested negative for *path_MLH1*, *path_MSH2* or *path_MSH6* known to be present in their family. This study can be considered for patients undergoing surveillance for LS
**Breast**	
Møller *et al*.^6^	In the PLSD, there is a relative incidence of breast cancer of 1·3 for *path_MLH1*, 1·2 for *path_MSH2* and 1·4 for *path_MSH6* carriers. In this cohort, the confidence intervals all include 1 and it is not possible to confirm a significant increase in breast cancer risk at this time

LS, Lynch syndrome; HNPCC, hereditary non‐polyposis colorectal cancer; PLSD, Prospective Lynch Syndrome Database.

### Conclusions and recommendations

- Consensus was not achieved for the statement ‘Surveillance for other cancers (than colorectal, endometrial and ovarian) should not be offered’.

## Question 4: what is the appropriate surgical treatment for colorectal cancer?

The risk of metachronous colorectal cancer after primary colonic cancer depends on which variant a carrier has and on the extent of surgery performed in managing the primary colonic cancer (how much colon is left to be at risk). There are no good data describing the risk of a second colonic cancer by gene and treatment of first cancer. Analyses in the PLSD^22^ suggested that the risk of a second colorectal cancer was determined stochastically: the risk of a second cancer was not substantially different from the risk of a first cancer by age, gene and gender. Without stratifying by gene and treatment of first colonic cancer (because numbers in the study did not allow substrata for analysis), a PLSD study^22^ reported that the risk of metachronous colorectal cancer by 70 years (starting from 40 years) after an earlier colonic cancer and despite regular colonoscopies was 36 (95 per cent c.i. 29 to 43·8) per cent. The numbers were likely to be conservative estimates for metachronous cancer, because a substantial proportion of these patients were treated at specialized centres and will have had extended colonic surgery. Three recent meta‐analyses and several independent studies evaluated the risk of metachronous colorectal cancer after colectomy for colonic cancer (*Table* *6*)^57–65^. No studies have reported the risk of a third colorectal cancer after subsequent colonic cancer. However, third metachronous colorectal cancers have been described, as has survival after these subsequent cancers^22^. There are no studies addressing the more invasive surgical option of extending colorectal surgery to a proctocolectomy at the occurrence of a first or metachronous colonic or rectal cancer. It is essential to evaluate the role of extended surgery prospectively and provide recommendations stratified by MMR gene in order to reduce the incidence of metachronous colorectal cancer occurring in *path_MMR* carriers.

**Table 6.** Studies describing surgical treatment for colorectal cancer in Lynch syndrome

Reference	Study outcomes
Anele *et al*.^57^	This report included six studies involving 871 patients who met the inclusion criteria (705 had segmental colectomy and 166 extended colectomy). The weighted mean follow‐up was 7·6 years. Patients with LS were four times more likely to develop metachronous CRC after segmental colectomy than those who had extended resection, despite regular endoscopic surveillance (1–2‐yearly)
Heneghan *et al*.^58^	This review included eight studies and 948 patients (780 had segmental colectomy and 168 extended colectomy) followed up for 8·9 years, but they did not exclude studies based on clinical criteria only for diagnosis of LS. The authors calculated the risk of metachronous CRC to be 3·7 times higher after segmental colectomy than extended resection. There were limitations regarding morbidity and mortality data in most of the original studies
Malik *et al*.^59^	This meta‐analysis identified 1389 patients with LS and HNPCC followed up for a mean of 8·4 years, with a mean age at onset of 45·5 years. A total of 1119 patients underwent segmental colectomies with an absolute risk of metachronous CRC of 22·4% at the end of follow‐up. The 270 patients who underwent extended colectomies had a metachronous CRC risk of 4·7%. Segmental colectomy was significantly associated with an increased RR of metachronous CRC (RR 5·12, 95% c.i. 2·88 to 9·11), although no significant association with mortality was identified (RR 1·65, 0·90 to 3·02)
Renkonen‐Sinisalo *et al*.^60^	The authors compared the outcomes of 242 genetically confirmed LS carriers who underwent either STC (98) or segmental colectomy (144), and were followed up for between 14·6 and 25 years. The cumulative risk of metachronous CRC after segmental colectomy was 47% compared with 7% after STC + ISA. Extended surgery also reduced the risk of subsequent abdominal surgery compared with segmental resection (10·9 *versus* 54·1%), but there was no difference in CRC‐specific survival. However, the majority of patients were *path_MLH1* carriers. For patients undergoing STC, 10–20 cm of sigmoid was left and an ISA performed
Kim *et al*.^61^	The cumulative risk of metachronous CRC was reported to be 20·4% in 10‐year follow‐up after segmental colectomy compared with 0% after extended surgery in this South‐Korean study of 106 patients with LS. No survival benefit of extended surgery was established
Parry *et al*.^62^	The authors reported a decrease in metachronous CRC risk for every 10 cm of bowel removed. The cumulative risk of metachronous CRC in 322 patients undergoing segmental colectomy was 16 (95% c.i. 10 to 25)% at 10 years, 41 (30 to 52)% at 20 years and 62 (50 to 77)% at 30 years. Of the 50 patients who underwent extensive colonic resection (STC + ISA or IRA) for their first colonic cancer, none were diagnosed with metachronous CRC over 414 person‐years of follow‐up
Stupart *et al*.^63^ Natarajan *et al*.^64^	Other retrospective comparative studies have reported a high incidence of metachronous CRC after segmental colectomy and a lower incidence after extended colectomy. STC with IRA or ISA decreases the risk of metachronous colonic cancer and provides easy endoscopic surveillance
You *et al*.^65^	This study of 201 extended resections (STC + ISA or TC + IRA) and 321 segmental colectomies for indications other than inflammatory bowel diseases reported a median of four daily bowel movements after ISA and five after IRA, despite considerable dietary restrictions (reported by 55·6 per cent) and medication use (19·6 per cent reported daily use). STC + ISA resulted in significantly less daytime and night‐time bowel movements than TC + IRA (*P* = 0·002) but no significant difference in quality‐of‐life measures (*P* = 0·16)

LS, Lynch syndrome; CRC, colorectal cancer; HNPCC, hereditary non‐polyposis colorectal cancer; RR, relative risk; STC, subtotal colectomy; ISA, ileosigmoidal anastomosis; IRA, ileorectal anastomosis; TC, total colectomy.

Extended surgery, either subtotal colectomy with ileosigmoidal anastomosis or total colectomy with ileorectal anastomosis, for primary colonic cancer in *path_MLH1* and *path_MSH2* carriers is preferable to reduce the risk of subsequent colorectal cancer, even though extended surgery has not been shown to improve overall survival. There is no substantial evidence to support extended surgery for *path_MSH6* and *path_PMS2* carriers. All surgical decision‐making should be personalized, based on the patient's age, gender, and expected functional outcome and priorities.

### Subquestion 1: should extended surgery for Lynch syndrome‐associated colonic cancer be recommended?

A largely retrospective body of evidence supports the extension of surgical resection in *path_MLH1* and *path_MSH2* carriers, but the risk of metachronous colorectal cancer in *path_MSH6* and *path_PMS2* carriers is not high enough to warrant an extended approach (*Table* *6*).

#### Conclusions and recommendations

- For a *path_MLH1* or *path_MSH2* carrier with a first colonic cancer, extended surgery with ileosigmoidal/ileorectal anastomosis is preferable to standard resection to reduce the risk of metachronous colorectal cancer.
- For a *path_MSH6* or *path_PMS2* pathogenic variant carrier with a first colonic cancer, standard/segmental colonic resection should be offered.
- For a *path_MMR* carrier with a metachronous colonic cancer, the surgical treatment can be extended surgery with ileorectal/ileosigmoidal anastomosis.
- A decision on extended surgery for colorectal cancer should not be based on deficient MMR immunohistochemistry (loss of MLH1, MSH2, MSH6 or PMS2) and BRAF staining/*MLH1* hypermethylation from the preoperative endoscopic biopsy only.

### Subquestion 2: how should surgery for rectal cancer in Lynch syndrome be performed?

Studies have reported metachronous cancer after rectal cancer in carriers^66^^,^^67^. Because the cumulative risk of a subsequent colonic cancer developing during surveillance is significant, extended surgery may be a personal preference, considering the higher degree of surgical morbidity associated with the procedure. Quality of life of patients with an ileoanal pouch may be comparable to that of patients with familial adenomatous polyposis rather than those with inflammatory bowel disease (IBD), because the worse overall outcome in the latter group is due to the underlying inflammatory condition. There is clear evidence that surgeons performing a high volume of procedures in high‐volume units (over 10 per year) achieve lower pouch failure rates as well as better pouch salvage than those with a lower throughput of these procedures^68^^,^^69^. Preferred surgical options by gene are shown in *Table* *7*.

**Table 7:** Surgical options for colorectal cancer in *path_MMR* carriers

	*Path_MLH1*	*Path_MSH2*	*Path_MSH6*	*Path_PMS2*	Additional risk factors
Primary colonic cancer of a known LS pathogenic variant carrier	Subtotal colectomy with ileosigmoidal (ileorectal) anastomosis	Subtotal colectomy with ileosigmoidal (ileorectal) anastomosis	Segmental colectomy (right, extended right or left colectomy)	Segmental colectomy (right, extended right or left colectomy)	Adenoma formation in a colonic segment other than the segment affected by colonic cancer Extended surgery such as colectomy with ileosigmoidal or ileorectal anastomosis. For young patients, consider more extensive surgery
Metachronous colonic cancer in a known LS pathogenic variant carrier who has undergone previous segmental colectomy	Subtotal colectomy with ileorectal anastomosis	Subtotal colectomy with ileorectal anastomosis	Subtotal colectomy with ileorectal anastomosis	Subtotal colectomy with ileorectal anastomosis	
Primary rectal cancer in a known LS pathogenic variant carrier	Anterior resection/APR	Anterior resection/APR	Anterior resection/APR	Anterior resection/APR	
Rectal cancer after previous colonic cancer surgery in a known LS pathogenic variant carrier	Proctectomy/proctocolectomy with ileoanal anastomosis with pouch or APR with permanent ileostomy	Proctectomy/proctocolectomy with ileoanal anastomosis with pouch or APR with permanent ileostomy	Proctectomy/proctocolectomy with ileoanal anastomosis with pouch or APR with permanent ileostomy		

LS, Lynch syndrome; APR, abdominoperineal resection.

#### Conclusions and recommendations

- For a *path_MMR* carrier, the surgical treatment of a primary rectal cancer (occurring as the first colorectal cancer) should be standard resection (anterior resection or abdominoperineal resection).
- In a young *path_MMR* carrier with a synchronous colorectal neoplasm or a personal preference, extended surgery can be considered.
- Ileoanal pouch surgery (in agreement with European CanCer Organisation guidelines for pouch surgery in ulcerative colitis) should be performed in highly specialized colorectal surgical units.

### Subquestion 3: should prophylactic bowel surgery be performed?

It is acknowledged that, even with the more favourable stage at diagnosis in patients under surveillance, there is a 9 per cent overall mortality rate among patients with LS in the 10 years after diagnosis of colorectal cancer. There is no documented procedure to identify and separate carriers at higher risk of colorectal cancer than the average by using age, gene and gender, nor those who might be at risk of not surviving a future colorectal cancer. There is no evidence base to support prophylactic colorectal surgery in *path_MMR* carriers.

In a study^70^ of patients with high‐grade dysplasia in endoscopic biopsies and a polyp that was not amenable to endoscopic removal, 22 of 165 polyps (13·3 per cent) had an invasive cancer on final pathology. In another study at the Mayo Clinic^71^, 133 of 750 unresectable polyps (17·7 per cent) harboured a malignancy, of which 23 per cent were lymph node‐positive. Although these studies were conducted without knowledge of the MMR status of the tumours, they indicate that high‐grade dysplasia on biopsy is a strong predictor of tumours harbouring invasive malignancy.

In patients with LS and concurrent IBD, it is unclear whether colorectal cancer risks are sufficiently increased for prophylactic colectomy to be indicated. In a small series^72^ of 12 patients with LS and concurrent IBD, four developed colorectal cancer in an early age. However, the series did not demonstrate a sufficiently increased risk of colorectal cancer to recommend prophylactic surgery.

#### Conclusions and recommendations

- For endoscopically non‐removable polyps with advanced histology, an oncological approach is recommended, as for gene‐specific treatment for carcinoma.
- Prophylactic colorectal surgery in the absence of neoplastic lesions in the colorectum is not recommended for *path_MMR* carriers based on their pathogenic variant‐related risk only.

## Question 5: what is the influence of lifestyle factors on the development of adenoma or colorectal cancer in Lynch syndrome?

The majority of studies showed positive associations between BMI and smoking and colorectal cancer, whereas some studies reported that alcohol consumption was positively associated with colorectal cancer. One study reported an inverse association between physical activity and colorectal cancer in *path_MMR* carriers (*Table* *8*)^73–89^. No lifestyle factor has been demonstrated to increase the risk of cancer specifically for one gender, or for carriers of pathogenic variants in a specific gene.

**Table 8:** Association studies between BMI, smoking, alcohol consumption, physical activity and colorectal cancer in Lynch syndrome

Reference	Study outcomes
Diergaarde *et al*.^73^ Botma *et al*.^74^ Campbell *et al*^75^ Movahedi *et al*.^76^ Win *et al*.^77^	These studies reported on the association between BMI and colorectal tumours in LS. A high BMI was associated with an increased risk of colorectal tumours in three publications^74^^,^^76^^,^^77^ another^75^ indicated an association in the same direction, and one^73^ did not show an association
Diergaarde *et al*.^73^ Kamiza *et al*.^78^ Watson *et al*.^79^ Winkels *et al*.^80^ Dashti *et al*.^81^	In analyses of alcohol consumption and colorectal tumour risk in LS, three studies^73^^,^^78^^,^^79^ did not observe an association, one^80^ demonstrated a possible increased risk, and one^81^ reported a significant increase in risk of colorectal tumours when alcohol consumption was high
Kamiza *et al*.^78^	The influence of physical activity on colorectal tumour risk has been investigated in only one study, which showed that being physically active is significantly associated with a decreased colorectal tumour risk in those with LS
Brand *et al*.^82^ Diergaarde *et al*.^73^ Pande *et al*.^83^ Watson *et al*.^79^ Winkels *et al*.^80^	The risk of colorectal tumours is significantly increased by smoking in LS
Diergaarde *et al*.^73^ Kamiza *et al*.^78^ Botma *et al*.^74^ Voskuil *et al*.^84^ Burn *et al*.^85^ Mathers *et al*.^86^ Jung *et al*.^87^ Chau *et al*.^88^ Heine‐Bröring *et al*.^89^	Different aspects of diet, such as dietary patterns^74^, and consumption of meat^73^^,^^78^^,^^84^, vegetables^73^^,^^78^, fruit^73^^,^^78^, fish^73^, dairy products^73^, dietary fibre^73^^,^^85^^,^^86^, dietary B vitamins^87^, dietary supplements^88^^,^^89^, tea^73^ and coffee^73^ have been investigated in relation to colorectal tumour risk in people with LS. None of these dietary factors, however, were evaluated in more than three publications

LS, Lynch syndrome.

### Conclusions and recommendations

- *Path_MMR* carriers should be advised that smoking and obesity increase the risk of colorectal cancer.
- *Path_MMR* carriers should be advised that alcohol consumption increases the risk of colorectal cancer.
- *Path_MMR* carriers should be advised that physical activity reduces the risk of colorectal cancer.

## Question 6: what is the role of acetylsalicylic acid (aspirin) in the management of Lynch syndrome?

An RCT^76,85,90^ showed that 600 mg acetylsalicylic acid daily for 2–4 years was well tolerated and reduced colorectal cancer incidence after 5 years following initiation of the treatment. A recently published study^91^ has shown that the benefit of prevention with acetylsalicylic acid persists into the second decade. Observational data in the general population suggest that lower doses may also be effective^92^^,^^93^. For bodyweight above average (70 kg), a higher dose than currently in clinical use for other indications may be required^94^.

### Conclusions and recommendations

- *Path_MMR* carriers should be advised that daily acetylsalicylic acid intake will reduce colorectal cancer risk.
- The recommended acetylsalicylic acid dose should be a minimum of 75–100 mg daily. This dose should be increased for people with above‐average body mass.

## Final conclusion

The recommendations from the EHTG and ESCP for identification of patients with LS, colorectal surveillance, surgical management of colorectal cancer, lifestyle and chemoprevention in LS that reached a consensus (at least 80 per cent) are presented here. The joint statement also endorsed the Manchester International Consensus Group recommendations for the management of gynaecological cancers in LS.

## Supplementary Material

znaa178_Supplementary_Data
