# Supplementary material for: European guidelines from the EHTG and ESCP for Lynch syndrome: an updated third edition of the Mallorca guidelines based on gene and gender
Source: Br J Surg. 2021 May 26;108(5):484–98. doi: 10.1002/bjs.11902 (PMC10364896; doi:10.1002/bjs.11902)
Supplement: znaa178_Supplementary_Data [file znaa178_supplementary_data.docx]

**European guidelines from the EHTG and ESCP for Lynch syndrome: an updated third edition of the Mallorca guidelines based on gene and gender**

T. T. Seppälä, A. Latchford, I. Negoi, A. Sampaio Soares, R. Jimenez-Rodriguez, L. Sánchez-Guillén, D. G. Evans, N. Ryan, E. J. Crosbie, M. Dominguez-Valentin, J. Burn, M. Kloor, M. von Knebel Doeberitz, F. J. B. van Duijnhoven, P. Quirke, J. R. Sampson, P. Møller and G. Möslein, on behalf of the European Hereditary Tumour Group (EHTG) and European Society of Coloproctology (ESCP)

**Appendix S1 Supporting information**

[Table S1: Clinical management summary. 3](#_Toc43921919)

[Table S2: Patient summary 6](#_Toc43921920)

[Table S3: Levels of quality of evidence by Grading of Recommendations, Assessment, Development and Evaluation – GRADE. 8](#_Toc43921921)

[PICOs (Patients, interventions, comparators, outcomes) 9](#_Toc43921922)

[Identification of Lynch syndrome (Ionut Negoi, Phil Quirke, Neil Ryan and Emma Crosbie) 9](#_Toc43921923)

[Questions: 9](#_Toc43921924)

[Patients: 9](#_Toc43921925)

[Interventions/comparator 9](#_Toc43921926)

[Outcomes 9](#_Toc43921927)

[Colorectal surveillance (Andrew Latchford) 9](#_Toc43921928)

[Questions: 9](#_Toc43921929)

[Patients 9](#_Toc43921930)

[Interventions/Comparators 9](#_Toc43921931)

[Outcomes 10](#_Toc43921932)

[Surveillance for other cancers (Antonio Sampaio Soares) 10](#_Toc43921933)

[Questions: 10](#_Toc43921934)

[Patients: 10](#_Toc43921935)

[Interventions/comparators 10](#_Toc43921936)

[Outcomes 10](#_Toc43921937)

[Colorectal surgery (Toni Seppälä and Louis Sánchez-Guillén) 10](#_Toc43921938)

[Questions: 10](#_Toc43921939)

[Patients: 11](#_Toc43921940)

[Interventions/comparators 11](#_Toc43921941)

[Outcomes 11](#_Toc43921942)

[Environment and lifestyle (Fränzel Dujnhoven) 11](#_Toc43921943)

[Questions: 11](#_Toc43921944)

[Patients: 11](#_Toc43921945)

[Interventions/comparators 11](#_Toc43921946)

[Outcomes 11](#_Toc43921947)

[Aspirin (John Burn and Rosa Jimenez Rodriguez) 12](#_Toc43921948)

[Questions: 12](#_Toc43921949)

[Patients: 12](#_Toc43921950)

[Interventions 12](#_Toc43921951)

[Outcomes: 12](#_Toc43921952)

[Table S4: The Delphi results. 13](#_Toc43921953)

[Table S5: Sensitivity and specificity of different diagnostic tests used to identify LS (modified after (1), an open access source) . 49](#_Toc43921954)

[References 50](#_Toc43921955)

[Appraisal of Guidelines for Research and Evaluation (AGREE) 51](#_Toc43921956)

[AGREE statements for “European Guidance from EHTG and ESCP for Lynch syndrome: an updated third edition of the “Mallorca Guidelines” based on Gene and Gender” 51](#_Toc43921957)

[Domain 1: Scope and Purpose 51](#_Toc43921958)

[Domain 2: Stakeholder involvement 51](#_Toc43921959)

[Domain 3: Rigour of development 52](#_Toc43921960)

[Domain 4: Clarity of presentation 53](#_Toc43921961)

[Domain 5: Applicability 53](#_Toc43921962)

[Domain 6: Editorial independence 54](#_Toc43921963)

[AGREE Reporting Checklist 55](#_Toc43921964)

# Table S1: Clinical management summary.

|  | *path_MLH1* | *path_MSH2* | *path_MSH6* | *path_PMS2* |
| --- | --- | --- | --- | --- |
| Typical IHC staining patterns | Loss of MLH1 and PMS2, similar to sporadic MLH1 hypermethylation | Loss of MSH2 and MSH6 | Isolated loss of MSH6 | Isolated loss of PMS2 |
| Unusual IHC staining patterns | Isolated loss of PMS2 (in 12%) | Isolated loss of MSH6 with normal expression of MSH2 | Heterogeneous loss of MSH6 (due to preopearative chemoradiotherapy in rectal cancer) | Loss of MSH6 and PMS2 |
|  |  | Loss of expression of MSH6 with cytoplasmic expression of MSH2 (caused by an EPCAM MSH2 fusion) |  |  |
| Colonoscopy surveillance | Strongly recommended | Strongly recommended | Strongly recommended | Strongly recommended |
| Age of colonoscopy surveillance onset | 25 years | 25 years | 35 years | 35 years |
| Interval of colonoscopies (no prior CRC) | No consensus between 2 and 3 years | No consensus between 2 and 3 years | No consensus between 2 and 3 years | 5 years may be considered |
| Interval of colonoscopies (prior CRC) | Bianneal | Bianneal | Bianneal | Bianneal |
| Management of first colon cancer | Subtotal colectomy with ileosigmoidal anastomosis (or ileorectal anastomosis) | Subtotal colectomy with ileosigmoidal anastomosis (or ileorectal anastomosis) | Hemicolectomy (segmental) based on standard principles | Hemicolectomy (segmental) based on standard principles |
| Management of first rectal cancer | Anterior resection with partial or total mesorectal excision | Anterior resection with partial or total mesorectal excision | Anterior resection with partial or total mesorectal excision | Anterior resection with partial or total mesorectal excision |
| Management of metachronous colon cancer | Subtotal colectomy with ileosigmoidal anastomosis (or ileorectal anastomosis) | Subtotal colectomy with ileosigmoidal anastomosis (or ileorectal anastomosis) | Subtotal colectomy with ileosigmoidal anastomosis (or ileorectal anastomosis) | Subtotal colectomy with ileosigmoidal anastomosis (or ileorectal anastomosis) |
| Risk-reducing total hysterectomy and salpingo-ovariectomy | Strongly recommended, no earlier than 35-40 years of age following completion of childbearing | Strongly recommended, no earlier than 35-40 years of age following completion of childbearing | Strongly recommended, no earlier than 35-40 years of age following completion of childbearing | No consensus |
| Surveillance for gastric cancer | No consensus for recommending / not recommending | No consensus for recommending / not recommending | No consensus for recommending / not recommending | No consensus for recommending / not recommending |
| Surveillance for small bowel cancer | No consensus for recommending / not recommending | No consensus for recommending / not recommending | No consensus for recommending / not recommending | No consensus for recommending / not recommending |
| Surveillance for pancreatic cancer | No consensus for recommending / not recommending | No consensus for recommending / not recommending | No consensus for recommending / not recommending | No consensus for recommending / not recommending |
| Surveillance for urinary tract cancers | No consensus for recommending / not recommending | No consensus for recommending / not recommending | No consensus for recommending / not recommending | No consensus for recommending / not recommending |
| Surveillance for breast cancer | No consensus for recommending / not recommending | No consensus for recommending / not recommending | No consensus for recommending / not recommending | No consensus for recommending / not recommending |
| Surveillance for prostate cancer | No consensus for recommending / not recommending | No consensus for recommending / not recommending | No consensus for recommending / not recommending | No consensus for recommending / not recommending |
| Aspirin use | Recommended | Recommended | Recommended | Recommended |
| Aspirin dose | 75-100 mg daily, increase if body mass greater than 70 kg | 75-100 mg daily, increase if body mass greater than 70 kg | 75-100 mg daily, increase if body mass greater than 70 kg | 75-100 mg daily, increase if body mass greater than 70 kg |

# Table S2: Patient summary

| Key points – Lynch syndrome |
| --- |
| - Inherited errors in the genes called *MLH1*, *MSH2*, *MSH6* and *PMS2* cause an increased cancer risk in multiple organs, most commonly bowel and womb cancer. This is Lynch syndrome. - If a person carries such an error, the risk of them passing it on to their child is 50%. - The risks of what cancer someone may develop changes depending on which gene the error is in. Therefore people may have their Lynch syndrome managed in different ways depending on their personal risk. - These guidelines summarise the evidence to support decision-making. |
| Key points – Identification |
| - Family history and things about your medical history are not accurate in identifying those people with Lynch syndrome - Therefore, all bowel cancers and womb cancers are recommended to be tested for Lynch syndrome - Evidence does not currently support routinely testing other cancers than bowel and womb in all individuals. |
| Key points – Surveillance for colorectal cancer |
| - Surveillance and removal of adenomas (polyps) by colonoscopies colonoscopies (camera inspection of the bowel) has been shown to reduce bowel cancer in Lynch syndrome compared to not performing colonoscopies. - Colonoscopies reduce death from bowel cancer compared to no screening by earlier diagnosis compared to no surveillance. - How often you should have a colonoscopy if you carry an error *in MLH1, MSH2, MSH6* is not clear. It would seem you can wait 3 years between colonoscopies. However your doctor may feel every 2 years is appropriate for you. However, if you have had a bowel cancer before, we recommend two-yearly colonoscopy. - *If you have an error in the PMS2* gene your risk of bowel cancer is lower. Also, the way in which your cancer grows is slower. Therefore 5-yearly colonoscopies are safe. - Colonoscopy surveillance should start at 25 years for errors in *MLH1* and *MSH2*, and at 35 for those with errors in *MSH6* and *PMS2*. - The recommended surveillance does not differ between men and women. |
| Key points – surgical management for colorectal cancer |
| - Life expectancy after bowel cancer is better than in general population, but after your first bowel cancer you have an increased risk of getting another bowel cancer. This risk can be reduced by surgically removing more bowel during the treatment of your first bowel cancer. This approach is called extended surgery. - Extended surgery at the time of the first bowel cancer is recommended if the error is in *MLH1* or *MSH2* genes. Evidence does not currently support extended surgery if the error is in *MSH6* and *PMS2* genes. However, you should talk to your treating surgeon about this as they will be able to personalise your surgery to your overall risk. - We do not recommend having surgery simply because you have an inherited gene error, only if you are having treatment for cancer. |
| Key points – life style and medication |
| - Smoking, obesity and alcohol consumption increase the risk of bowel cancer in Lynch syndrome - Physical activity reduces bowel cancer risk in Lynch syndrome. - Daily aspirin of at least 75 to 100 mg reduces cancer risk in Lynch syndrome. The dose should be individually increased with physicians advise for persons with body weight above average. |

# Table S3: Levels of quality of evidence by Grading of Recommendations, Assessment, Development and Evaluation – GRADE.

| Quality of evidence | Definition |
| --- | --- |
| High | Further research is very unlikely to change our confidence in the estimate of effect   - Several high-quality studies with consistent results - In special cases: one large, high-quality multi-center trial |
| Moderate | Further research is likely to have an important impact on our confidence in the estimate of effect and may change the estimate   - One high-quality study - Several studies with some limitations |
| Low | Further research is very likely to have an important impact on our confidence in the estimate of effect and is likely to change the estimate   - One or more studies with severe limitations |
| Very low | Any estimate of effect is very uncertain   - Expert opinion - No direct research evidences - One or more studies with severe limitations |

# PICOs (Patients, interventions, comparators, outcomes)

## **Identification of Lynch syndrome (Ionut Negoi, Phil Quirke, Neil Ryan and Emma Crosbie)**

### Questions:

- Are clinical criteria useful for finding LS?
- Is universal screening effective to find LS?

### Patients:

- All colorectal cancer patients
- All endometrial cancer patients
- All upper urinary tract patients (UTUC)

### Interventions/comparator

- Clinical criteria guided vs. universal screening
- Universal MMR/MSI screening vs. no universal testing
- Germline multipanel testing vs. no testing

### Outcomes

- Increased identification of LS

## **Colorectal surveillance (Andrew Latchford)**

### Questions:

- What is the optimal colorectal surveillance protocol for LS?
- should colonoscopy surveillance be performed?
- should chromoendoscopy/virtual chromoendoscopy be performed?
- what is the appropriate colonoscopy surveillance interval?
- What is appropriate colonoscopy surveillance after surgical colorectal resection for cancer?
- At what age should the surveillance be initiated?

### Patients

- Pathogenic variant carriers of *MLH1, MSH2, MSH6, PMS2, EPCAM*

### Interventions/Comparators

- Colonoscopy surveillance vs. no surveillance
- Chromoendoscopy vs. white-light endoscopy

### Outcomes

- Primary measure: colorectal cancer mortality
- Secondary measures:
  - decrease of CRC lifetime incidence
  - detection of dysplastic lesions
  - complications of surveillance and treatment
  - costs of surveillance and treatment

## **Surveillance for other cancers (Antonio Sampaio Soares)**

### Questions:

- Should OGDs be performed for gastric and duodenal cancers?
- Should surveillance by MRI or EUS be performed for pancreatic cancer?
- Should surveillance for urothelial cancer be performed by urine cell analysis?
- Should gastroscopies be performed?
- Should mammographies be performed?
- Should PSA screening be performed?

### Patients:

- *MLH1, MSH2, MSH6, PMS2, EPCAM* carriers

### Interventions/comparators

- Pancreaticobiliary MRI vs. no screening
- EUS vs. no screening
- Urine cells vs. no screening
- Cell free DNA vs. no screening
- PSA vs. no screening

### Outcomes

- Primary measure: cancers-specific mortality
- Secondary measures:
  - risks of surveillance and treatment
  - costs of surveillance and treatment

## **Colorectal surgery (Toni Seppälä and Louis Sánchez-Guillén)**

### Questions:

- What is the appropriate surgical treatment for CRC?
- Should extended surgery be performed for CRC?
- Should prophylactic surgery be performed to prevent CRC in healthy path_MMR carriers?
- how should surgery for LS rectal cancer be performed?
- to what extent should surgery be done based on preoperative dMMR from histology?
- Should decision on extended surgery for CRC be based on dMMR immunohistochemistry only?

### Patients:

- *MLH1, MSH2, MSH6, PMS2, EPCAM* carriers

### Interventions/comparators

- prophylactic surgery vs. endoscopy surveillance
- extended surgery vs. standard surgery

### Outcomes

- Primary measure: metachronous CRC incidence
- Secondary measures:
  - Overall mortality
  - CRC mortality
  - abdominal surgery rate
  - bowel function
  - Risk of performing extended surgery on patients with normal risk

## **Environment and lifestyle (Fränzel Dujnhoven)**

### Questions:

Is there evidence for dietary factors?

Is there evidence for smoking?

Is there evidence for alcohol consumption?

Is there evidence for obesity?

Is there evidence for physical activity?

### Patients:

- *MLH1, MSH2, MSH6, PMS2, EPCAM* carriers

### Interventions/comparators

- Smoking vs. no smoking
- Hight body mass index (BMI) vs. low BMI
- High consumption of alcohol vs. low consumption of alcohol
- High physical activity vs. low physical activity
- High consumption of dietary factors vs. low consumption of dietary factors

### Outcomes

- CRC incidence

## **Aspirin (John Burn and Rosa Jimenez Rodriguez)**

### Questions:

- Does aspirin reduce risk of CRC?
- Does aspirin reduce risk of other cancers?
- What is the recommended dose of aspirin to prevent cancer?

### Patients:

- *MLH1, MSH2, MSH6, PMS2, EPCAM* carriers

### Interventions

- Aspirin 600 mg vs. no aspirin
- Aspirin 300 mg vs. no aspirin
- Aspirin 100/75 mg vs. no aspirin

### Outcomes:

- Primary measure: CRC incidence
- Secondary measures:
  - Extracolonic cancer incidence
  - Complications of regular and long-term aspirin intake

# Table S4: The Delphi results.

| Question | Round 1  (Total responses = 57) | Round 2  (total responses = 64) | Round 3  (Total responses = 18) |
| --- | --- | --- | --- |
| Q1: What is your current expertise related to Lynch Syndrome? | Gastroenterologist – 14.29%  Geneticist – 25.00%  Genetic counselor – 8.93%  Surgeon – 19.64%  Gynecologist – 1.79%  Pathologist – 1.79%  Other clinical specialty – 1.79%  Trainee – 3.57%  Nurse – 3.57%  Patient / family member – 0.00%  Patient advocate – 0.00%  Researcher (none of the above) – 19.64% |  |  |
| CLINICAL CRITERIA |  |  |  |
| Q2: In terms of specificity and sensitivity, are the Amsterdam Criteria and Revised Bethesda Criteria to be implemented to guide tumor testing in Lynch Syndrome? | Yes – 23.91%  No – 76.09% | Yes – 13.79%  No – 86.21% |  |
| UNIVERSAL SCREENING |  |  |  |
| Q3: In terms of specificity and sensitivity, should universal (unselected) tumor testing by immunohistochemical staining of MMR proteins (MLH1, MSH2, MSH6 and PMS2) followed by BRAF IHC and/or MLH1 hypermethylation testing be recommended in colorectal cancer? | Yes – 95.65% |  |  |
| Q4: In terms of specificity and sensitivity, should universal tumor testing by immunohistochemical staining of MMR proteins (MLH1, MSH2, MSH6 and PMS2) followed by BRAF IHC and/or *MLH1* hypermethylation testing be recommended in endometrial cancer? | Yes – 84.44%  No – 8.89%  No, systematic reflex testing is better – 6.67% | Yes – 90.91% |  |
| Q5: In terms of specificity and sensitivity, should colorectal cancer immunohistochemical tumor testing by staining of MMR proteins be recommended to be undertaken on preoperative tumor biopsies? | Yes – 59.09%  Yes, but only if supported by a feature related to Lynch syndrome (family history, multiple primary tumors) – 27.27%  No – 13.64% |  |  |
|  |  | Is MMR IHC as accurate on pre-operative biopsy as post-operative resection specimen?  Yes – 81.36% |  |
| Q6: Which cancers of Lynch Syndrome tumor spectrum should MMR IHC universal tumor testing cover without age restriction? | Colorectal cancer – 86.36%  Endometrial cancer – 81.82%  Ovarian cancer – 18.18%  Urothelial cancer (all urinary tract) – 27.27%  Upper urinary tract urothelial cancer – 20.45%  Gastric cancer – 15.91%  Pancreatic cancer – 15.91%  Biliary tract cancer – 13.64%  None – 11.36% |  |  |
|  |  | Should universal MMR IHC be performed on upper urinary tract cancers?  Yes – 77.36% |  |
|  |  |  | Universal screening can be performed on upper urinary tract carcinomas to identify Lynch Syndrome. Weak recommendation based on low quality evidence.  Agree – 73.33%  Disagree – 26.67% |
| Q7: Which cancers of Lynch Syndrome tumor spectrum should MMR IHC universal tumor testing cover with age restriction? | Colorectal cancer – 35.00%  Endometrial cancer – 40.00%  Ovarian cancer – 42.50%  Urothelial cancer (all urinary tract) – 40.00%  Upper urinary tract urothelial cancer – 40.00%  Gastric cancer – 47.50%  Pancreatic cancer – 40.00%  Biliary tract cancer – 37.50%  None – 22.50% |  |  |
| Q8: Is germline multipanel sequencing without preselection via MMR immunohistochemistry a recommendable approach if finances were covered by the healthcare system? | Yes – 57.14% |  |  |
|  |  | Is germline multipanel sequencing without preselection via MMR immunohistochemistry, recommended in individuals with CRC, if resources allow?  Yes – 59.65% |  |
| NEED FOR COLONOSCOPY SURVEILLANCE |  |  |  |
| Q9: Should colonoscopy surveillance in Lynch Syndrome be performed to reduce mortality and incidence of colorectal cancer in *MLH1* or *MSH2* pathogenic variant carriers? | Yes – 100% |  |  |
| Q10: Should colonoscopy surveillance in Lynch Syndrome be performed to reduce mortality and incidence of colorectal cancer in *MSH6* pathogenic variant carriers? | Yes – 100% |  |  |
| Q11: Should colonoscopy surveillance in Lynch Syndrome be performed to reduce mortality and incidence of colorectal cancer in *PMS2* pathogenic variant carriers? | Yes – 82.93% |  |  |
| COLONOSCOPY INTERVAL |  |  |  |
| Q12: For *MLH1* or *MSH2* pathogenic variant carriers without previous CRC, what is the appropriate colonoscopy surveillance interval to reduce mortality and incidence of CRC? | < 1 year – 2.44%  1 year – 12.20%  1-2 years – 48.76%  2 years – 17.07%   - 1. Years – 14.63%   3 Years – 2.44%  > 3 years – 0.00%  Other – 2.44% | 1 year – 17.65%  2 years – 68.63%  3 years – 13.73% |  |
| Q13: For a *MSH6* pathogenic variant carrier without previous CRC, what is the appropriate colonoscopy surveillance interval to reduce mortality and incidence of CRC? | < 1 year – 0.00%  1 year – 2.38%   - 1. years – 33.33%   2 years – 33.33%  2-3 Years – 19.05%  3 Years – 9.52%  > 3 years – 0.00%  Other – 2.38% | 1 year – 6%  2 years – 66%  3 years – 28% |  |
|  |  |  | For *MLH1, MSH2* and *MSH6* pathogenic variant carriers, 2-yearly colonoscopy surveillance is recommended. Strong recommendation based on moderate quality evidence. Further supportive data are required before extending the surveillance interval to 3 years can be recommended.  Agree – 75%  Disagree – 25% |
| Q14: For a *PMS2* pathogenic variant carrier without previous CRC, what is the appropriate colonoscopy surveillance interval to reduce mortality and incidence of CRC? | < 1 year – 0.00%  1 year – 2.38%  1-2 years – 16.67%   1. years – 19.05%   2-3 Years – 19.05%  3 Years – 14.29%  > 3 years – 14.29%  Other – 14.29% | 1 year – 13.73%  2 years – 41.18%  3 years – 45.1% | For *PMS2* pathogenic variant carriers, 5-yearly surveillance may be considered. Weak recommendation based on low quality evidence.  Agree – 80%  Disagree – 20% |
| Q15: For *MLH1* or *MSH2* pathogenic variant carriers with previous CRC and segmental colectomy, what is the appropriate colonoscopy surveillance interval to reduce mortality and incidence of CRC? | < 1 year – 2.27%  1 year – 29.55%  1-2 years – 47.73%   1. years – 6.82%   2-3 Years – 4.55 %  3 Years – 2.27%  > 3 years – 0.00%  Other – 6.82% | 1 year – 38.18%  2 years – 56.36%  3 years – 5.45% |  |
| Q16: For a *MSH6* pathogenic variant carrier with previous CRC and segmental colectomy, what is the appropriate colonoscopy surveillance interval to reduce mortality and incidence of CRC? | < 1 year – 0.00 %  1 year – 22.73%   - 1. years – 45.45%   2 years – 13.64%  2-3 Years – 9.09%  3 Years – 4.55%  > 3 years – 0.00%  Other – 4.55% | 1 year – 21.74%  2 years – 67.39%  3 years – 10.87% |  |
| Q17: For a *PMS2* pathogenic variant carrier with previous CRC and segmental colectomy, what is the appropriate colonoscopy surveillance interval to reduce mortality and incidence of CRC? | < 1 year – 2.38%  1 year – 19.05%  1-2 years – 26.19%   1. years – 14.29%   2-3 Years – 21.43%  3 Years – 4.76%  > 3 years – 7.14%  Other – 4.76% | 1 year – 9.3%  2 years – 58.14%  3 years – 32.56% |  |
| Q18: For a *MLH1* or *MSH2* pathogenic variant carrier with previous CRC and a subtotal colectomy (with ileosigmoidal or ileorectal anastomosis), what is the appropriate endoscopy surveillance interval to reduce mortality and incidence of CRC? | < 1 year – 2.38%  1 year – 28.57%  1-2 years – 33.33%  2 years – 19.05%   - 1. Years – 7.14%   3 Years – 2.38%  > 3 years – 0.00%  Other – 7.14% | 1 year – 25.53%  2 years – 68.09%  3 years – 6.38% |  |
| Q19: For a *MSH6* pathogenic variant carrier with previous CRC and a subtotal colectomy (with ileosigmoidal or ileorectal anastomosis), what is the appropriate endoscopy surveillance interval to reduce mortality and incidence of CRC? | < 1 year – 2.44%  1 year – 17.07%  1-2 years – 34.15%  2 years – 21.95%   - 1. Years – 12.20%   3 Years – 4.88%  > 3 years – 0.00%  Other – 7.32% | 1 year – 22.22%  2 years – 62.22%  3 years – 15.56% |  |
| Q20: For a *PMS2* pathogenic variant carrier with previous CRC and a subtotal colectomy (with ileosigmoidal or ileorectal anastomosis), what is the appropriate endoscopy surveillance interval to reduce mortality and incidence of CRC? | < 1 year – 0.00%  1 year – 20.00%  1-2 years – 27.50%  2 years – 10.00%   - 1. Years – 15.00%   3 Years – 10.00%  > 3 years – 10.00%  Other – 7.50% | 1 year – 20%  2 years – 51.11%  3 years – 28.89% |  |
|  |  |  | For LS patients with previous CRC and segmental colectomy, biennial colonoscopies should be performed. Strong recommendation based on moderate quality evidence.  Agree – 87.50%  Disagree – 12.50% |
|  |  |  | For LS patients with previous CRC and subtotal colectomy, biennial rectosigmoidoscopies should be performed. Strong recommendation based on moderate quality evidence.  Agree – 87.50%  Disagree – 12.50% |
|  |  |  | There is no evidence at the moment to support different surveillance colonoscopy intervals for males and females. Strong recommendation based on moderate quality evidence.  Agree – 100% |
| CHROMOENDOSCOPY |  |  |  |
| Q21: Should chromoendoscopy be performed to reduce mortality and incidence of CRC in Lynch syndrome? | Yes – 61.76% |  |  |
|  |  |  | Virtual chromoendoscopy can be considered but may be less effective than chromoendoscopy. Strong recommendation based on moderate quality evidence.  Agree – 70%  Disagree – 30% |
|  |  |  | Chromoendoscopy is equivalent to high definition white light endoscopy in specialist centres. It may be an adjunct to be considered in the absence of high definition endoscopy or in centres with lower adenoma detection rates. Weak recommendation based on strong quality evidence.  Agree – 91.67%  Disagree – 8.33% |
| AGE OF ONSET OF THE COLONOSCOPY SURVEILLANCE |  |  |  |
| Q22: For a *MLH1* pathogenic variant carrier, at what age should surveillance colonoscopies be initiated to reduce mortality and incidence of colorectal cancer? | At 20 years of age – 24.39%  At 25 years of age – 60.98%  At 30 years of age – 4.88%  At 35 years of age – 2.44%  At 40 years of age – 2.44%  After first cancer – 0.00%  Based on family history – 2.44%  Other – 2.44% |  |  |
| Q23: For a *MSH2* pathogenic variant carrier, at what age should surveillance colonoscopies be initiated to reduce mortality and incidence of colorectal cancer? | At 20 years of age – 26.83%  At 25 years of age – 56.10%  At 30 years of age – 7.32%  At 35 years of age – 2.44%  At 40 years of age – 2.44%  After first cancer – 0.00%  Based on family history – 2.44%  Other – 2.44% |  |  |
|  |  | For a *MLH1* or *MSH2* pathogenic variant carrier, at what age should surveillance colonoscopies be initiated to reduce mortality and incidence of colorectal cancer?  20 years – 22.22%  25 years – 77.78% |  |
| Q24: For a *MSH6* pathogenic variant carrier, at what age should surveillance colonoscopies be initiated to reduce mortality and incidence of colorectal cancer? | At 20 years of age – 12.50%  At 25 years of age – 15.00%  At 30 years of age – 40.00%  At 35 years of age – 22.50%  At 40 years of age – 5.00%  After first cancer – 0.00%  Based on family history – 2.50%  Other – 2.50% | 25 years – 30.61%  35 years – 63.27%  40 years – 6.12% |  |
|  |  |  | For a *MLH1* or *MSH2* pathogenic variant carrier, surveillance colonoscopies should be initiated at the age of 25 years. Moderate recommendation based on low quality evidence.  Agree – 93.75%  Disagree – 6.25% |
| Q25: For a *PMS2* pathogenic variant carrier, at what age should surveillance colonoscopies be initiated to reduce mortality and incidence of colorectal cancer? | At 20 years of age – 7.69%  At 25 years of age – 10.26%  At 30 years of age – 30.77%  At 35 years of age – 17.95%  At 40 years of age – 10.26%  At 50 years – 7.69%  After first cancer – 0.00%  Based on family history – 12.82%  Other – 2.56% | 25 years – 21.15%  35 years – 59.62%  45 years – 19.23% |  |
|  |  |  | For a *MSH6* or *PMS2* pathogenic variant carrier, surveillance colonoscopies should be initiated at the age of 35 years. Moderate recommendation based on low quality evidence.  Agree – 93.33%  Disagree – 6.67% |
|  |  |  | Age of onset of surveillance should not be stratified by gender. Moderate recommendation based on moderate quality evidence.  Agree – 87.50%  Disagree – 12.50% |
|  |  |  | Age of onset of surveillance colonoscopy should be stratified according to genotype. Strong recommendation based on moderate quality evidence.  Agree – 100% |
| COLONOSCOPY QUALITY |  |  |  |
|  |  |  | If bowel preparation is not entirely adequate, a repeat procedure at 1 year is recommended. If the bowel preparation is completely inadequate or if the examination is incomplete then an immediate repeat colorectal surveillance procedure should be requested. Weak recommendation based on very low-quality evidence (expert opinion).  Agree – 84.62%  Disagree – 15.38% |
|  |  |  | If polypectomy has been performed, a repeat procedure at 1 year may be considered. Weak recommendation based on very low quality evidence.  Agree – 61.54%  Disagree – 38.46% |
| OTHER SURVEILLANCE |  |  |  |
|  |  |  | Surveillance for other cancers (than colorectal, endometrial and ovarian) should not be offered. Moderate recommendation based on moderate quality evidence.  Agree – 68.75%  Disagree – 31.25% |
| SURGERY – FIRST COLON CANCER |  |  |  |
| Q26: For a female *MLH1* pathogenic variant carrier, should extended surgery for colon cancer be recommended? | Yes – 54.05%  No – 24.32%  Other – 21.62% |  |  |
| Q27: For a male *MLH1* pathogenic variant carrier, should extended surgery for colon cancer be recommended? | Yes – 59.46%  No – 21.62%  Other – 18.92% |  |  |
| Q28: For a female *MSH2* pathogenic variant carrier, should extended surgery for colon cancer be recommended? | Yes – 55.26%  No – 23.68%  Other – 21.05% |  |  |
| Q29: For a male *MSH2* pathogenic variant carrier, should extended surgery for colon cancer be recommended? | Yes – 65.79%  No – 15.79%  Other – 18.42% |  |  |
|  |  | For *MLH1* and *MSH2* pathogenic variant carriers, provided expected functional outcome is acceptable, should extended surgery for colon cancer be recommended?  Yes – 66.67% |  |
|  |  |  | For a *MLH1* or *MSH2* pathogenic variant carrier with first colon cancer, extended surgery with ileosigmoidal/ileorectal anastomosis should be preferred over standard resection to reduce metachronous CRC risk. Strong recommendation based on moderate quality evidence.  Agree – 82.35%  Disagree – 17.65% |
| Q30: For a female *MSH6* pathogenic variant carrier, should extended surgery for colon cancer be recommended? | Yes – 35.14%  No – 45.95%  Other – 18.92% |  |  |
| Q31: For a male *MSH6* pathogenic variant carrier, should extended surgery for colon cancer be recommended? | Yes – 32.43%  No – 45.95%  Other – 21.62% |  |  |
| Q32: For a female *PMS2* pathogenic variant carrier, should extended surgery for colon cancer be recommended? | Yes – 27.03%  No – 62.16%  Other – 10.81% |  |  |
| Q33: For a male *PMS2* pathogenic variant carrier, should extended surgery for colon cancer be recommended? | Yes – 27.03%  No – 62.16%  Other – 10.81% |  |  |
|  |  | For *PMS2* pathogenic variant carriers, provided expected functional outcome is acceptable, should extended surgery for colon cancer be recommended?  Yes – 11.11% |  |
|  |  |  | For a *MSH6* or *PMS2* pathogenic variant carrier with first colon cancer, standard/segmental colonic resection can be preferred. Weak recommendation based on moderate quality evidence.  Agree – 80%  Disagree – 20% |
| Q35: For a female *MLH1* or *MSH2* pathogenic variant carrier, what should the recommended extent of colonic resection for first colon cancer be in order to reduce mortality and incidence of metachronous CRC? | Standard segmental resection – 24.32%  Subtotal colectomy – 56.76%  Proctocolectomy – 0.00%  Other – 18.92% |  |  |
| Q36: For a male *MLH1* or *MSH2* pathogenic variant carrier, what should the recommended extent of colonic resection for first colon cancer be in order to reduce mortality and incidence of metachronous CRC? | Standard segmental resection – 24.32%  Subtotal colectomy – 56.76%  Proctocolectomy – 0.00%  Other – 18.92% |  |  |
|  |  | For a *MLH1* or *MSH2* pathogenic variant carrier, should extended colorectal resection for primary rectal cancer be recommended in order to reduce mortality and incidence of metachronous CRC?  Yes – 50% |  |
| Q37: For a female *MSH6* pathogenic variant carrier, what should the recommended extent of colonic resection for first colon cancer be in order to reduce mortality and incidence of metachronous CRC? | Standard segmental resection – 50.00%  Subtotal colectomy – 33.33%  Proctocolectomy – 0.00%  Other – 16.67% |  |  |
| Q38: For a male *MSH6* pathogenic variant carrier, what should the recommended extent of colonic resection for first colon cancer be in order to reduce mortality and incidence of metachronous CRC? | Standard segmental resection – 50.00%  Subtotal colectomy – 33.33%  Proctocolectomy – 0.00%  Other – 16.67% |  |  |
|  |  | For a *MSH6* pathogenic variant carrier, should extended colorectal resection for primary rectal cancer be recommended in order to reduce mortality and incidence of metachronous CRC?  Yes – 28.57% |  |
| Q39: For a female *PMS2* pathogenic variant carrier, what should the recommended extent of colonic resection for first colon cancer be in order to reduce mortality and incidence of metachronous CRC? | Standard segmental resection – 66.67%  Subtotal colectomy – 22.22%  Proctocolectomy – 0.00%  Other – 11.11% |  |  |
| Q40: For a male *PMS2* pathogenic variant carrier, what should the recommended extent of colonic resection for first colon cancer be in order to reduce mortality and incidence of metachronous CRC? | Standard segmental resection – 66.67%  Subtotal colectomy – 22.22%  Proctocolectomy – 0.00%  Other – 11.11% |  |  |
|  |  | For a *PMS2* pathogenic variant carrier, should extended colorectal resection for primary rectal cancer be recommended in order to reduce mortality and incidence of metachronous CRC?  Yes – 7.69% |  |
| SURGERY - PROPHYLACTIC |  |  |  |
| Q34: Should prophylactic colorectal surgery without neoplastic lesions in the colorectum be suggested in Lynch Syndrome based on the mutation risk only? | Yes – 2.63%  No – 97.37% |  |  |
| SURGERY – RECTAL CANCER |  |  |  |
| Q41: For a female *MLH1* or *MSH2* pathogenic variant carrier, what should the extent of colorectal resection for primary rectal cancer be in order to reduce mortality and incidence of metachronous CRC? | Standard procedure – 55.56%  Proctocolectomy – 16.67%  Other – 27.78% |  |  |
| Q42: For a male *MLH1* or *MSH2* pathogenic variant carrier, what should the extent of colorectal resection for primary rectal cancer be in order to reduce mortality and incidence of metachronous CRC? | Standard procedure – 52.78%  Proctocolectomy – 16.67%  Other – 30.56% |  |  |
| Q43: For a female *MSH6* pathogenic variant carrier, what should the extent of colorectal resection for primary rectal cancer be in order to reduce mortality and incidence of metachronous CRC? | Standard procedure – 61.11%  Proctocolectomy – 16.67%  Other – 22.22% |  |  |
| Q44: For a male *MSH6* pathogenic variant carrier, what should the extent of colorectal resection for primary rectal cancer be in order to reduce mortality and incidence of metachronous CRC? | Standard procedure – 61.11%  Proctocolectomy – 16.67%  Other – 22.22% |  |  |
| Q45: For a female *PMS2* pathogenic variant carrier, what should the extent of colorectal resection for primary rectal cancer be in order to reduce mortality and incidence of metachronous CRC? | Standard procedure – 75.00%  Proctocolectomy – 11.11%  Other – 13.89% |  |  |
| Q46: For a male *PMS2* pathogenic variant carrier, what should the extent of colorectal resection for primary rectal cancer be in order to reduce mortality and incidence of metachronous CRC? | Standard procedure – 75.00%  Proctocolectomy – 11.11%  Other – 13.89% |  |  |
|  |  |  | Primary rectal cancer of all path_MMR carriers must be managed by a rectal resection by standard oncological total mesorectal excision (TME). Strong recommendation based on high quality of evidence.  Agree – 84.2%  Disagree – 15.38% |
|  |  |  | For a path_MMR carrier, the surgical treatment of a primary rectal cancer (as first colorectal cancer) should be standard resection (anterior resection or abdominoperineal resection). Strong recommendation based on moderate quality evidence.  Agree – 91.67%  Disagree – 8.33% |
|  |  |  | In a young age onset rectal cancer of a MMR pathogenic variant carrier with a synchronous neoplasia or personal preference, extended surgery can be considered. Weak recommendation based on very low quality evidence.  Agree – 85.71%  Disagree – 14.29% |
|  |  |  | Ileoanal pouch surgery (in agreement with the ECCO guidelines for pouch surgery in ulcerative colitis) should be performed in highly specialized colorectal surgical units. Moderate recommendation based on low quality evidence.  Agree – 91.67%  Disagree – 8.33% |
| SURGERY – METACHRONOUS CANCER |  |  |  |
| Q47: What should be the extent of colonic resection for a metachronous colon cancer? | Standard segmental resection – 17.14%  Subtotal colectomy – 74.29%  Proctocolectomy – 0.00%  Other – 8.57% |  |  |
|  |  | In patients with Lynch syndrome, what should be the extent of colonic resection for a metachronous colon cancer to reduce the risk of further metachronous cancer?  Standard segmental – 11.11%  Subtotal colectomy – 88.89% |  |
|  |  |  | For a path_MMR carrier with a metachronous colon cancer, the surgical treatment can be extended surgery with ileorectal/ileosigmoidal anastomosis. Weak recommendation based on very low quality evidence.  Agree – 93.33%  Disagree – 6.67% |
| SURGERY - MISCELLANEOUS |  |  |  |
| Q48: Should extended colorectal surgery be based on dMMR immunohistochemical (loss of MLH1, MSH2, MSH6 or PMS2) and wild type BRAF staining from preoperative endoscopic biopsy? | Yes – 17.14%  No – 62.86%  Other – 20.00% | Yes – 6.38%  No – 93.62% |  |
| Q49: What should be the extent of colorectal resection for endoscopically unremovable high grade dysplastic colon lesion (but not verified CRC) in a young (e.g. 45-year-old) individual with a verified MMR pathogenic variant? | Standard segmental resection – 39.39%  Subtotal colectomy – 36.36%  Proctocolectomy – 0.00%  Local excision / resection – 3.03%  Other – 21.21% |  |  |
| Q50: What should be the extent of colorectal resection for endoscopically unremovable high grade dysplastic colon lesion (but not verified CRC) in an elderly (e.g. 70-year-old) individual with a verified MMR pathogenic variant? | Standard segmental resection – 75.76%  Subtotal colectomy – 9.09%  Proctocolectomy – 0.00%  Local excision / resection – 6.06%  Other – 9.09% |  |  |
|  |  | Is the surgical management of endoscopically irresectable high grade dysplasia the same as for CRC for each genotype, provided expected function is acceptable?  Yes – 76.92%  No – 23.08% |  |
|  |  |  | In case of endoscopically non-removable polyp with advanced histology, an oncological approach is recommended, with no difference to gene-specific approach of a carcinoma. Strong recommendation based on moderate quality evidence.  Agree – 92.86%  Disagree – 7.14% |
| LIFESTYLE FACTORS |  |  |  |
| Q51: Patients with Lynch Syndrome should be advised, that smoking increases the risk of colorectal cancer. | Yes – 100% |  |  |
| Q52: Patients with LS should be advised, that obesity increases the risk of colorectal cancer | Yes – 100% |  |  |
| Q53: Patients with Lynch Syndrome should be advised, that alcohol consumption increases the risk of colorectal cancer. | Yes – 81.82% |  |  |
| Q54: Patients with LS should be advised, that physical activity reduces the risk of colorectal cancer | Yes – 89.74% |  |  |
| Q55: The evidence for dietary factors affecting colorectal cancer risk in Lynch Syndrome is insufficient for recommendations. | Strongly agree – 2.94%  Agree – 41.18%  Neither agree nor disagree – 14.71%  Disagree – 32.35%  Strongly disagree – 8.82% |  |  |
|  |  | Is there evidence that dietary factors affect CRC risk in Lynch syndrome?  Yes – 63.41% |  |
| Q56: The effect of gender on environmental and lifestyle factors has not yet been systematically studied and should be an area of future collaborative studies | Strongly agree – 27.03%  Agree – 59.46%  Neither agree nor disagree – 10.81%  Disagree – 2.70%  Strongly disagree – 0.00% |  |  |
| Q57: Should patients with LS be recommended to take Aspirin to reduce incidence of cancer? | Yes – 68.42%  No – 7.89%  Other – 23.68% | Yes – 72.92%  No – 27.08% |  |
|  |  |  | Patients with *path_MMR* should be advised that there is a high probability that daily aspirin will reduce their cancer risk. Weak recommendation based on moderate quality evidence.  Agree – 100% |
| Q58: If aspirin is recommended, what dose should be taken? | 75 mg – 14.29%  100 mg – 22.86%  150 mg – 11.43%  300 mg – 11.43%  600 mg – 2.86%  Other – 37.14% |  |  |
|  |  | Until CAPP3 is published, what dose of Aspirin should be recommended?  75-100 mg: 34.29%  300 mg: 45.71%  600 mg: 20% |  |
|  |  |  | The recommended aspirin dose should be a minimum of 75-100 mg daily. This dose should be increased for people with above average body mass. Weak recommendation based on low quality evidence.  Agree – 92.86%  Disagree – 7.14% |
| Q59: If Aspirin is recommended, data are insufficient to recommend a duration of therapy? | Strongly agree – 17.14%  Agree – 57.14%  Neither agree nor disagree – 17.14%  Disagree – 8.57%  Strongly disagree – 0.00% | Agree – 78.05%  Disagree – 21.95% |  |
| Q60: Whether the effect of Aspirin varies by gene and gender has not yet been systematically studied and should be an area of future collaborative studies | Strongly agree – 27%  Agree – 73%  Neither agree nor disagree –  Disagree –  Strongly disagree – |  |  |
| Q61: Is there sufficient evidence to recommend implementation of  immunoscores in order to improve the identification of LS? | Yes – 17.65%  No – 70.59%  Other – 11.76% |  |  |
| Q62: With the existing evidence, is immune checkpoint blockade therapy (PD-1 blockade / vaccines) an option in the management of advanced solid LS-associated cancers? | Yes – 80.00%  No – 8.57%  Other – 11.43% | Yes – 100% |  |
| Q63: Should MMR status of a tumor be systematically tested in order to adapt adjuvant therapy? | Yes – 91.67%  No – 2.78%  Other – 5.56% |  |  |
| Q64: Does deficient MMR of the tumor translate to a lack of benefit from 5-FU chemotherapy? | Yes – 69.70%  No – 6.06%  Other – 24.24% |  |  |
|  |  | Is single agent 5-FU chemotherapy recommended for patients with a cancer displaying deficient MMR?  Yes – 17.14%  No – 82.86% |  |
| Q65: Does MMR status affect response or survival in patients receiving oxaliplatin treatment? | Yes – 37.50%  No – 25.00%  Other – 37.50% |  |  |
| Q66: I am interested in being involved in the Delphi process | Yes – 94.74% |  |  |
| Q67: I will attend the EHTG meeting in Nice in September 2018 | Yes – 89.74% |  |  |
| Q68: I wish to be involved in the final Delphi voting round (envisaged shortly after the Nice meeting, perhaps involving a teleconference) | Yes – 94.74% |  |  |

# Table S5: Sensitivity and specificity of different diagnostic tests used to identify LS (modified after (1), an open access source) .

| **First author, publishing year** | **Continent, Included patients** | **Clinical criteria (Se/Sp)** | **IHC (Se/Sp)** | **MSI (Se/Sp)** | **BRAF (Se/Sp)** | **Gene sequencing (Se/Sp)** |
| --- | --- | --- | --- | --- | --- | --- |
| **Chen Y-E, 2016 (2)** | Asia, CRC patients | NA | 83% / 88.8% | 76-89% / 90.2% | 69% / 99% | 99.5% / 99.96% |
| **Severin F, 2015 (3)** | Europe, CRC patients | Amsterdam II: 27.2% / 97.9% | 86.8% / 91% | 76-89% / 90.7% | 70% / 99% | 99.5% / 100% |
| **Gallego CJ, 2015 (6)** | USA, Patients sent for genetic evaluation | NA | 83% / 89% | NA | 69% / 99% | 99.9% / 99.9% |
| **Barzi A, 2015 (5)** | USA, CRC patients | Amsterdam II: 22% / 98% | 83% / 89% | 85% / 90% | NA | 100% / 100% |
| **Dinh TA, 2011 (6)** | USA, general population | NA | 83% / 88.8% | NA | NA | 90% / 99.97% |
| **Ladabaum U, 2011 (7)** | USA, CRC patients | Amsterdam II: 22% / 98% | 83% / 89% | 85% / 90.2% | NA | NA |
| **Mvundura M, 2010** (50) | USA, CRC patients | NA | 83% / 88.8% | 89% / 90.2% | 69% / 99% | 99.5% / 99.96% |
| **Ramsey SD, 2003 (8)** | USA, CRC patients | Bethesda: 70% / 85% | NA | 91% / 93% | NA | 87% / 99.5% |
| Se – Sensitivity, Sp – Specificity, IHC – immunohistochemistry, MSI – microsatellite instability, BRAF - B-Raf proto-oncogene, NA – not available | | | | | | |

# References

1. Di Marco M, DAndrea E, Panic N, Baccolini V, Migliara G, Marzuillo C, et al. Which Lynch syndrome screening programs could be implemented in the “real world”? A systematic review of economic evaluations. Genet Med. 2018 Oct;20(10):1131–44.

2. Chen Y-E, Kao S-S, Chung R-H. Cost-Effectiveness Analysis of Different Genetic Testing Strategies for Lynch Syndrome in Taiwan. Toland AE, editor. PLOS ONE. 2016 Aug;11(8):e0160599–e0160599.

3. Severin F, Stollenwerk B, Holinski-Feder E, Meyer E, Heinemann V, Giessen-Jung C, et al. Economic evaluation of genetic screening for Lynch syndrome in Germany. Genet Med. 2015 Oct;17(10):765–73.

4. Gallego CJ, Shirts BH, Bennette CS, Guzauskas G, Amendola LM, Horike-Pyne M, et al. Next-Generation Sequencing Panels for the Diagnosis of Colorectal Cancer and Polyposis Syndromes: A Cost-Effectiveness Analysis. J Clin Oncol. 2015 Jun;33(18):2084–91.

5. Barzi A, Lenz H-J, Quinn DI, Sadeghi S. Comparative effectiveness of screening strategies for colorectal cancer. Cancer. 2017 May;123(9):1516–27.

6. Dinh TA, Rosner BI, Atwood JC, Boland CR, Syngal S, Vasen HFA, et al. Health Benefits and Cost-Effectiveness of Primary Genetic Screening for Lynch Syndrome in the General Population. Cancer Prev Res (Phila Pa). 2011 Jan;4(1):9–22.

7. Ladabaum U, Wang G, Terdiman J, Blanco A, Kuppermann M, Boland CR, et al. Strategies to Identify the Lynch Syndrome Among Patients With Colorectal Cancer. Ann Intern Med. 2011 Jul;155(2):69–69.

8. Ramsey SD, Burke W, Clarke L. An economic viewpoint on alternative strategies for identifying persons with hereditary nonpolyposis colorectal cancer. Genet Med. 2003;

# Appraisal of Guidelines for Research and Evaluation (AGREE)

## AGREE statements for “European Guidance from EHTG and ESCP for Lynch syndrome: an updated third edition of the “Mallorca Guidelines” based on Gene and Gender”

### Domain 1: Scope and Purpose

**1 -** This guideline covers the screening, diagnosis and management of Lynch syndrome and is targeted for patients with this condition. Its aim is to gather the most up to date evidence on the management of Lynch syndrome.

**2 –** The guideline covers the following questions: “How can we improve the identification of LS?”, “What is the optimal colorectal surveillance protocol for LS?”, “What is the effectiveness of surveillance for other cancers?”, “What is the appropriate surgical treatment for colorectal cancer?”, “What is the influence of lifestyle factors on the development of adenoma or colorectal cancer in LS?”, ” What is the role of acetylsalicylate in the management of LS?”.

**3 –** This guideline is directed to patients of both sexes and all age groups where the diagnosis of Lynch syndrome is suspected or confirmed. The recommendations are directed to all patients with the condition, irrespective of severity or comorbidities.

### Domain 2: Stakeholder involvement

**4 –** *[author name, affiliation, role and background]* The guidance was developed by Toni T. Seppälä (colorectal surgeon, project lead, Department of Surgery, Helsinki University Hospital and University of Helsinki, Helsinki, Finland, Department of Surgical Oncology, Johns Hopkins Hospital, Baltimore, Maryland, USA), Andrew Latchford (gastroenterologist, Department of Cancer and Surgery, Imperial College London and St Mark’s Hospital, London North West Healthcare NHS Trust, United Kingdom), Ionut Negoi (colorectal surgeon, literature search and recommendation write up, Department of Surgery, Emergency Hospital of Bucharest, Carol Davila University of Medicine and Pharmacy Bucharest, Romania), Antonio Sampaio Soares (surgeon, literature search and recommendation write up, Hospital prof. Dr. Fernando Fonseca, EPE, Lisbon, Portugal), Rosa Jimenez-Rodriguez (colorectal surgeon, literature search and recommendation write up, UGC Cirugía General y Aparato Digestivo, Hospital Universitario Virgen del Rocío, Seville, Spain), Luis Sánchez-Guillén (colorectal surgeon, literature search and recommendation write up, Colorectal Unit, Hospital Universitario y Politécnico La Fe, Valencia Hospital La Fe, University of Valencia, Valencia, Spain.), D Gareth Evans (geneticist, literature search and recommendation write up, Manchester Centre for Genomic Medicine, Division of Evolution and Genomic Sciences, University of Manchester, Manchester University Hospitals NHS Foundation Trust, Manchester, UK), Neil Ryan (gynaecologist, literature search and recommendation write up, Division of Cancer Sciences, Faculty of Biology, Medicine and Health, University of Manchester, St Mary's Hospital, Manchester, UK.), Emma J. Crosbie (gynaecologist, Division of Cancer Sciences, Faculty of Biology, Medicine and Health, University of Manchester, St Mary's Hospital, Manchester, UK.), Mev Dominguez-Valentin (geneticist, Department of Tumour Biology, The Norwegian Radium Hospital, Part of Oslo University Hospital, Oslo, Norway), John Burn (geneticist, Institute of Human Genetics, Newcastle upon Tyne, UK), Matthias Kloor (immuno-oncology scientist, Department of Applied Tumour Biology, Institute of Pathology, University Hospital Heidelberg and Cooperation Unit Applied Tumour Biology, German Cancer Research Center (DKFZ), Heidelberg, Germany), Magnus von Knebel Doeberitz (immuno-oncology scientist, Department of Applied Tumour Biology, Institute of Pathology, University Hospital Heidelberg and Cooperation Unit Applied Tumour Biology, German Cancer Research Center (DKFZ), Heidelberg, Germany), Fränzel J.B. van Duijnhoven (professor of Human Nutrition and Health Research, Division of Human Nutrition and Health, Wageningen University & Research, Wageningen, the Netherlands), Phil Quirke (pathologist, critical review of the manuscript, Pathology and Tumour Biology, Leeds Institute of Cancer and Pathology, University of Leeds, Leeds, UK), Julian R. Sampson (geneticist, Institute of Medical Genetics, Division of Cancer and Genetics, Cardiff University School of Medicine, Heath Park, Cardiff CF14 4XN, Cardiff, UK), Pål Møller (geneticist, Department of Tumour Biology, The Norwegian Radium Hospital, Part of Oslo University Hospital, Oslo, Norway and University of Witten/Herdecke, Germany), Gabriela Möslein (colorectal surgeon, project development and lead, Center for Hereditary Tumours, Bethesda Hospital, Duisburg and University of Witten/Herdecke, Germany)

**5 –** Patient involvement was appraised by involving representatives from two patient associations of Lynch syndrome patients on Delphi process.

**6 –** This guideline is directed to patients with LS and their medical providers. It should guide clinical decision-making and inform a shared discussion of the management options available for each patient.

### Domain 3: Rigour of development

**7 –** A PICO-model structure (Patients, Intervention, Comparison, Outcome) was created for each area of interest, based upon previously published template questions. A systematic literature search was performed using the Pubmed database and the Cochrane Database of Systematic Reviews and manual searches of relevant articles up until November 2018. The following MeSH terms were used: "hereditary nonpolyposis colorectal cancer"[All Fields] OR "Lynch syndrome"[All Fields], identifying 3893 articles. The titles were screened and relevant articles written in English were reviewed and graded into high, moderate, low and very low for the level of evidence according to GRADE criteria (<http://www.gradeworkinggroup.org/> ; Supplementary Table S3).

**8 –** Studies on Lynch syndrome patients were included with no design exclusions. Only papers written in English were included.

**9 –** Studies included were graded into high, moderate, low and very low for the level of evidence according to GRADE criteria (<http://www.gradeworkinggroup.org/>; Supplementary Table S3).”

**10 –** Suggestions for guidance statements emerging from the literature review were formulated and tested through Delphi model–based voting rounds. The first Delphi round took place as an online questionnaire on SurveyMonkey® (www.surveymonkey.co.uk) at week 38, 2018. The stakeholders were identified from the membership of EHTG to provide multidisciplinary expertise. The statements were thereby revised for a second Delphi round via live voting taking place at week 39, 2018, among participants at the third annual meeting of EHTG in Nice, France. The statements were revised again, and a third Delphi round was conducted by SurveyMonkey® voting at week 32–34, 2019, again among multidisciplinary stakeholders identified by EHTG. The most recent results from the Prospective Lynch Syndrome Database (PLSD) studies were disclosed to all participants before voting but were only published later 10. The threshold for reaching consensus at the Delphi votes was set at 80%.

**11 –** Each recommendation includes the supporting data and relevant references on the benefits and harms of the interventions and results from a balance between both.

**12 –** Each recommendation includes the supporting data and relevant references on the benefits and harms of the interventions and results from a balance between both.

**13 –** This guidance has been reviewed by an external methodologist, Jos Klejnen, Professor of Systematic Reviews in Health Care at the School for Public Health and Primary Care (CAPHRI), Maastricht University, The Netherlands.

**14 –** These guidelines will be available online and work as a “living guideline”, which will be updated every 6 months when new data is available.

### Domain 4: Clarity of presentation

**15 –** present in the executive summary and supplementary material

**16 –** present in the executive summary and supplementary material

**17 –** The executive summary clearly identifies the key recommendations.

### Domain 5: Applicability

**18 –** This guidance has been developed to be used by frontline clinicians caring for patients with LS. Barriers to adoption have been considered for all recommendations and included in the discussion.

The recommendations arising from the literature search were assessed with the Delphi methodology with a diverse group of stakeholders. Recommendations with an agreement over 80% were included in the final guidance with one exception (guidance between 2 or 3 year interval was not consented but there was a consensus of the regular colonoscopy surveillance).

**19 –** This guidance covers the key recommendations in the executive summary and includes an in-depth analysis in the online material. Visuals to facilitate understanding the guidance are included in the form of algorithms. Furthermore, it includes a link to an online calculator of the cancer risks designed to inform shared decision-making with the patients.

**20 –** Cost effectiveness analysis was a factor considered in the creation of the recommendations when it was available, as evidenced in the literature review that was performed. Otherwise, clinical outcomes were used to inform recommendations.

**21 –** Implementation of the guideline will be assessed in the ongoing research efforts of both the EHTG and ESCP. The next updates of the PLSD will collect information on outcomes of the healthcare including the adherence to these recommendations.

### Domain 6: Editorial independence

**22 –** None of the funding bodies influenced the content of the recommendation.

**23 –** All the authors of this guidance have completed the International Committee of Medical Journal Editors form for the disclosure of conflicts of interest.

## AGREE Reporting Checklist
